# Preclinical Research in Glycogen Storage Diseases: A Comprehensive Review of Current Animal Models

**DOI:** 10.3390/ijms21249621

**Published:** 2020-12-17

**Authors:** Aitana Almodóvar-Payá, Mónica Villarreal-Salazar, Noemí de Luna, Gisela Nogales-Gadea, Alberto Real-Martínez, Antoni L. Andreu, Miguel Angel Martín, Joaquin Arenas, Alejandro Lucia, John Vissing, Thomas Krag, Tomàs Pinós

**Affiliations:** 1 Mitochondrial and Neuromuscular Disorders Unit, Vall d’Hebron Institut de Recerca, Universitat Autònoma de Barcelona, 08035 Barcelona, Spain; aitana.alpa@gmail.com (A.A.-P.); Monicaazucena.villarreal@e-campus.uab.cat (M.V.-S.); alberto.real2289@gmail.com (A.R.-M.); 2 Centro de Investigación Biomédica en Red de Enfermedades Raras (CIBERER), 28029 Madrid, Spain; NLuna@santpau.cat (N.d.L.); gnogalga7@gmail.com (G.N.-G.); mamcasanueva@h12o.es (M.A.M.); joaquin.arenas@salud.madrid.org (J.A.); 3 Laboratori de Malalties Neuromusculars, Institut de Recerca Hospital de la Santa Creu i Sant Pau, Universitat Autònoma de Barcelona, 08041 Barcelona, Spain; 4 Grup de Recerca en Malalties Neuromusculars i Neuropediàtriques, Department of Neurosciences, Institut d’Investigacio en Ciencies de la Salut Germans Trias i Pujol i Campus Can Ruti, Universitat Autònoma de Barcelona, 08916 Badalona, Spain; 5 EATRIS, European Infrastructure for Translational Medicine, 1081 HZ Amsterdam, The Netherlands; toniandreu@eatris.eu; 6 Mitochondrial and Neuromuscular Diseases Laboratory, 12 de Octubre Hospital Research Institute (i+12), 28041 Madrid, Spain; 7 Faculty of Sport Sciences, European University, 28670 Madrid, Spain; alejandro.lucia@universidadeuropea.es; 8 Copenhagen Neuromuscular Center, Department of Neurology, Rigshospitalet, University of Copenhagen, DK-2100 Copenhagen, Denmark; john.vissing@regionh.dk (J.V.); thomas.krag@regionh.dk (T.K.)

**Keywords:** glycogen storage diseases, animal models, therapy

## Abstract

GSD are a group of disorders characterized by a defect in gene expression of specific enzymes involved in glycogen breakdown or synthesis, commonly resulting in the accumulation of glycogen in various tissues (primarily the liver and skeletal muscle). Several different GSD animal models have been found to naturally present spontaneous mutations and others have been developed and characterized in order to further understand the physiopathology of these diseases and as a useful tool to evaluate potential therapeutic strategies. In the present work we have reviewed a total of 42 different animal models of GSD, including 26 genetically modified mouse models, 15 naturally occurring models (encompassing quails, cats, dogs, sheep, cattle and horses), and one genetically modified zebrafish model. To our knowledge, this is the most complete list of GSD animal models ever reviewed. Importantly, when all these animal models are analyzed together, we can observe some common traits, as well as model specific differences, that would be overlooked if each model was only studied in the context of a given GSD.

## 1. Animal Models in Research

Due to the difficulty of studying disease mechanistically using invasive techniques in patients, animal models have been used for decades to better understand the altered characteristics and pathways involved in the different diseases, thereby contributing to the medical knowledge and advancement [1]. In the nineteenth century, French scientists François Magendie and Claude Bernard established the systematic experimentation in animals as a definitive characteristic of research in physiology [2,3]. In the twentieth century, the use of animal models for medical purposes incremented significantly, and while there is still some debate about the ethics of their use, at this moment, animal experimentation has become the most extended and accurate method of demonstrating biological significance [4]. In this regard, the usage of animal models for experimentation has majorly contributed to our understanding of the physio-pathologic processes of human diseases and to develop commonly used medicines such as vaccines or antibiotics [1]. Claude Bernard described the role of the pancreas in digestion using rabbits, dogs and other animals [5], Albert Sabin used monkeys to develop the polio vaccine [6], insulin was discovered in the 1920s in dogs [7], and the majority of antibiotics are tested on animals prior to trial in humans [8,9]. In the 1980s there was an explosion in the field of genetics and scientists became progressively capable to manipulate the genome of mice, with the subsequent arrival of transgenic mice carrying additional genetic material, or conversely knock-out mice in which genetic material is deleted [4]. In truth, diabetes research has been built on genetically modified murine models. Lately, our capacity to modify the mouse genome has become increasingly sophisticated with the emergence of new techniques and strategies such as: methods promoting to turn on (or off) gene transcription in vivo using tetracycline or tamoxifen (TM) activated systems, tissue-specific methods of knocking-out genes (e.g., the Cre-Lox system), or techniques for identifying or withdrawing entire cell linages in vivo via fluorescent protein and diphtheria toxin receptor knock-in mice, respectively [4].

In recent years, organoids (i.e., three-dimensional miniature cellular structures mimicking internal organs) have been derived from human stem cells. Useful information has been gleaned from the study of these organoids. However, these will be useful supplements but not replacements for animal model research, which often requires the complexity of whole body or organ for proper evaluation [10]. Today, around 20 million animals are used in biomedical research, being rodent models (mouse and rat) the most common [11]. In the present review, we perform an in-depth description of all the animal models existing up to date for the study of glycogen storage diseases (GSD), as well as the different therapeutic approaches that have been evaluated in these models.

## 2. Glycogen Storage Diseases

### 2.1. Introduction

GSD are a group of disorders characterized by a defect in gene expression of specific proteins (or a specific subunit of a multimeric enzyme complex) involved in glycogen breakdown or synthesis, frequently resulting in the increase of the glycogen stores in different tissues (primarily the liver and skeletal muscle, although other tissues such as the peripheral and central nervous system, the myocardium and renal tubules may also be affected) [12,13,14,15,16]. In GSD, hypoglycemia is a clear sign that the liver is affected, whilst exercise intolerance, fatigue, muscle cramps and/or contractures, and muscle weakness clearly suggest glycogen accumulation in skeletal muscle [12,13,14,15,16]. These unique diseases differ from each other in age of symptoms onset, morbidity and mortality, and there is actually substantial clinical phenotypic variation even within each disease. Prognosis might vary depending on the specific mutation of the enzyme, with some patients presenting mild forms of the disease, with few symptoms or minor exercise intolerance, whereas other forms might cause death within the first year of life [16]. Although it might be clinically helpful to divide GSD into those affecting primarily either the liver or the skeletal muscle tissue, respectively, GSD are typically classified according to the specific enzyme deficiency (Table 1).

Early diagnosis is key for patients to enter treatment if available or adjust diet to improve quality of life, reduce the damage in organs due to glycogen accumulation and increase the lifespan [12,13,14,15,16]. Nutritional interventions have been implemented to improve the symptoms and signs of GSDs even though they do not work for all the GSDs and do not represent a cure for these diseases [16]. For this reason, several different animal models have been found to naturally present spontaneous mutations and others have been developed and characterized in order to further understand the physiopathology of these diseases and as a useful tool to evaluate potential therapeutic strategies (Table 2).

### 2.2. Animal Models

A complete list of all GSD animal models can be found in Table 2. A list of the different mouse models for the distinct GSD and their main characteristics can be found in Table 3, while a list of the different GSD naturally occurring models and their characteristics can be found in Table 4.

### 2.3. Types of GSD

#### 2.3.1. GSD Type 0 (GSD-0; Aglycogenosis)

##### Introduction

GSD-0 (GSD-0a: OMIM #240600; GSD-0b OMIM #611556) is an autosomal recessive disorder caused by the absence or deficiency of the glycogen synthase enzyme (GS) first described by Lewis and collaborators in 1963 [20]. In mammals there are two GS isoforms: muscle GS (GS-M; encoded by the *GYS1* gene), and liver GS (GS-L; encoded by the *GYS2* gene). *GYS1* is ubiquitously expressed, but most abundantly in skeletal and cardiac muscle, while *GYS2* expression is restricted to the liver [229]. There are two different types of GSD-0:

(a) **GSD-0a** is caused by pathogenic mutations in both alleles of the *GYS2* gene (in humans located to chromosome 12p12.2). Deficiency in GS-L leads to a significant reduction in liver glycogen content as dietary carbohydrate is metabolized to ultimately produce lactate rather than being stored as glycogen [16]. During infancy patients frequently present with early morning somnolence and fatigue, sometimes accompanied with convulsions related to ketotic hypoglycemia. Hyperglycemia, glycosuria, and hyperlactic acidemia commonly occur during the post-pandrial period, which alternate with hypoglycemia and hyperketonemia during fasting. GSD-0a symptoms are those directly related with hypoglycemia and include nausea, vomiting, lethargy, pale appearance, and sometimes, seizures episodes in the morning before breakfast. Although in some patients there might be some degree of developmental delay, most affected children present normal development and are not cognitively affected. Short stature and osteopenia have also been reported [12,16]. The relatively benign course of this disease in comparison to other hepatic glycogenosis might be explained as both gluconeogenesis and fatty acid oxidation are not affected [12].

***Treatment.*** Treatment consist primarily on dietary management as protein-rich meals every 4 h rapidly diminish the symptoms, as well as night ingestion of uncooked cornstarch in low fat milk. The increase in protein ingestion during meals provides the substrate for gluconeogenesis, while a reduction of carbohydrates in the diet helps to reduce postprandial hyperglycemia, glycosuria, and hyperlactic acidemia [16].

(b) **GSD-0b** is caused by pathogenic mutations in both alleles of the *GYS1* gene (in humans located to chromosome 19q13.3). Only two families with GSD-0b have been reported [21,22]. Patients presented with a clear reduction of glycogen levels in the skeletal and cardiac-muscle, increased cardiac mass, predominance of oxidative fibers in skeletal muscle tissue, severely reduced capacity to sustain muscle work and normal to high glucose clearance [21]. Additionally, these two families showed case of spontaneous abortions, stillbirth and early death [22].

##### Mouse Model for GSD-0b

The ***Gys1^-/-^ mouse model*** was developed by inserting long terminal repeat (LTR) and β-Geo, β-galactosidase–neomycin phosphotransferase fusion gene sequences in the intron upstream of exon 12 of the *Gys1* gene. In this model there was a clear decrease in the number of pups per litter, and of the number of *Gys1^-/-^* mice obtained (i.e., only 10%, well below the 25% predicted by Mendelian genetics from crosses of heterozygotes), as most of them (90%) died between 11.5 days postcoitum (dpc) and 18.5 dpc due to abnormal cardiac development [106]. Additionally, *Gys1^-/-^* embryos were also affected with venous and pulmonary congestion. Those mice that survived were completely devoid of glycogen in the skeletal muscle and heart, and both tissues did not show major morphological or histological abnormalities, although the heart had remarkable fibrosis at old ages (12 to 16 months) [106,107]. At 3 months of age, male and female *Gys1^-/-^* mice were 10 and 5% lighter, respectively, than their wild-type (WT) controls, with reduced fat mass and epididymal fat pads in the former [108]. In the skeletal muscle (gastrocnemius), there was an increase in the number of slow, oxidative type I fibers compared with WT littermates whereas the amount of fast glycolytic type IIb fibers decreased and the proportion of type IIb oxidative glycolytic fibers was not significantly different [108]. In terms of exercise capacity, *Gys1^-/-^* mice exhibited normal physical activity and exercise performance under fed state. However, after overnight fasting, there was a trend to a reduced exercise endurance [107].

#### 2.3.2. GSD Type I (GSD-I; von Gierke Disease)

##### Introduction

GSD-I (GSD-Ia: OMIM #232200; GSD-Ib: OMIM #232220) is an autosomal recessive disorder first described by von Gierke in 1929 [23], caused by **glucose-6-phosphatase enzyme** (**G6Pase**) deficiency. G6Pase is mainly found in liver, kidney and the mucosa of the small intestine and plays a key role in glucose homeostasis as it catalyzes the hydrolysis of glucose-6-phosphate (G6P) to glucose and inorganic phosphate (Pi) in the final step of gluconeogenesis and glycogenolysis [24]. GSD-I has an annual incidence of 1/100,000 births representing approximately the 30% of all hepatic GSD [25]. The absence of G6Pase activity causes an impairment in endogenous glucose production resulting in lack of increase of blood glucose levels in response to positive gluco-regulatory stimuli; as a consequence, this condition is characterized by fasting hypoglycemia, high glycogen depots within liver and kidney, as well as lactic acidosis, hypertriglyceridemia and hyperuricemia as a consequence of shunting of G6P into alternative metabolic pathways [15,26,27,28]. The G6Pase enzyme is a hydrophobic protein constituted by 9 transmembrane helices localized in the endoplasmic reticulum (ER) and functions as a complex constituted by the catalytic (G6P_C_; encoded by *G6PC* gene; 17q21.31), and the transporter subunit (G6P_T_, encoded by *SLC37A4* gene; 11q.23.3). The G6P_C_ is localized on the luminal side of the ER, hence the G6P substrate must be translocated from the cytoplasm into the ER lumen by the G6PT in order to be hydrolyzed [230,231]. The main clinical features of GSD-I include hepatomegaly, nephromegaly and/or hypoglycemic seizures in childhood. Affected children often have doll-like faces due to adipose tissue deposition, short stature and poor musculature. Two different types of GSDI have been described:

(a) **GSD-Ia** is caused by a defect of the G6P_C_ subunit and constitutes the most prevalent form of GSD-I, representing approximately 80% of all cases [29,30]. Patients present with fasting hypoglycemia, hepatomegaly, nephromegaly, hyperlipidemia, hyperuricemia, lactic acidemia and growth retardation [27,232,233]. Main symptoms and signs (particularly in the morning or before feeding) are tremors, irritability, hyperventilation, cyanosis, apnea, convulsions, sweating, pallor, cerebral edema or dysfunction, coma, finally resulting in death [12]. Nose bleeding frequently occurs due to impaired platelet function, as well as rickets, anemia and diarrhea worsens with age [12]. Liver shows an enlargement at birth or shortly thereafter, causing an abdominal protuberance due to the massive hepatomegaly. Furthermore, steatosis can develop and xanthoma might be found on extensor surfaces as elbows and knees, sometimes accompanied with enlargement of kidneys. Hyperuricemia occurs due to both decreased renal clearance and increased production of urate. Hyperlipidemia occurs as a result of increased synthesis of triglycerides, VLDL and LDL, and decreased peripheral lipolysis [23]. Patients might develop long-term complications, such as hepatic adenomas, renal dysfunction, osteoporosis, gout or pancreatitis [12].
(b) **GSD-Ib** is caused by a defect of G6P_T_ subunit [29,30]. Patients with GSD-Ib have similar symptoms to those of patients with GSD-Ia. In addition, they have other characteristic alterations, mainly neutropenia and neutrophil dysfunction, which predispose to recurrent infections usually during the first year of life [12,16]. Affected children are prone to suffer recurrent oral mucosal ulceration, gingivitis, rapidly progressive periodontal disease, otitis and severe infections [16]. The *SLC37A4* gene is expressed in hematopoietic progenitor cells, which might explain the presence of neutropenia and frequent infections [12]. Neutropenia and neutrophil dysfunction usually leads to inflammatory bowel disease (Crohn-like) with fever, diarrhea and perioral and anal ulcers, a very common symptom of GSD-Ib [12,16]. Long-term complications can also develop, such as kidney disease in the form of renal calculi and progressive renal disease, splenomegaly and hepatocellular adenoma (HCA) [12].
(c) The existence of other subtypes (**types Ic and Id**) has not yet been confirmed [234,235].

***Treatment:*** Treatment of GSD-I consists primarily of maintaining normal blood glucose levels through dietary management [28]. Generally, nasogastric infusion of glucose or ingestion of uncooked cornstarch is employed [236]. This treatment alleviates the metabolic abnormalities associated with GSD-I and improve disease prognosis. Nevertheless, the main cause of the pathology is not solved, and long-term complications such as gout, hepatic adenomas with risk of malignancy, osteoporosis, intestinal disorders, progressive renal disease, delayed puberty, short stature and pulmonary hypertension can develop in adult patients [25,28,29,30,237,238,239].

##### Animal Models for GSD-Ia

(a) Canine model

***The Maltese canine model*** was the first animal model reported for GSD-Ia [109]. GSD-Ia was described in two Maltese dog littermates aged 47 days. Puppies presented with failure to thrive, hypoglycemia, hepatomegaly, necropsy and poor body condition. Liver was severely enlarged and pale, and kidneys were also pale. Hepatocytes had vacuoles with large amounts of glycogen and lipid droplets, and kidney tubular epithelium was mildly to moderately vacuolated. Furthermore, biochemical analysis showed that G6P levels were markedly reduced in liver and kidney. Puppies suffered premature death at 5–7 weeks of age [109]. Genetic basis was analyzed and a missense mutation was found in codon 121 with a guanine-to-cytosine mutation resulting in a substitution of methionine by isoleucine (p.121M>I) in the G6P_C_ protein [110].

Due to the intrinsic characteristics of this canine model such as the small size of their breed and litter size as well as the low survival of the newborns, carrier Maltese dogs were crossbred with Beagles in order to establish a breeding colony. Affected puppies presented with postnatal growth retardation, tremors, weakness, neurologic signs when hypoglycemic, and progressive hepatomegaly. Biochemical analysis revealed increased liver glycogen content and isolated markedly reduced G6Pase enzyme activity in liver and kidney. Liver showed hepatocellular vacuolization with distended clear hepatocytes and kidneys also had glycogen vacuoles [116].

(b) Mouse models

The ***G6pc^-^/^-^ mouse model*** was obtained by replacing *G6pc* exon 3 and its associated introns with a neomycin (neo) cassette, resulting in a null *G6pc* locus *in vivo*, leading to a total knockout (KO) mice [117]. Phenotypically, *G6pc^-^/^-^* mice showed slower growth with significantly less weight as well as hypoglycemia, hyperlipidemia and hyperuricemia during post-natal development; however, plasma lactate concentrations were not increased (in contrast to what occur in patients). Hepatomegaly and nephromegaly were not observed in newborn *G6pc^-^/^-^* mice but were developed at 5 days of age, with marked glycogen accumulation in both tissues. Glycogen accumulation was progressive and markedly pronounced by 20 to 26 days. Electron microscopy analysis also revealed the presence of prominent lipid vacuoles in the hepatocytes [117]. Additionally, the *G6pc*^-^/^-^ mice also had dysplasia of the cartilage at 5 days; the lesions occurred at the knee joint and epiphyseal plate of the long bones. By 16 days, cartilage grew in irregular plates with gross deformity of the joints [117]. The majority of the *G6pc*^-^/^-^ mice survived weaning, but less than 12% survived beyond 5 weeks of age [117]. Necropsy analysis of an 87 day-old *G6pc*^-^/^-^ mouse showed pulmonary edema, severe hepatic lipidosis, and glycogen storage in liver and kidneys. The liver showed marked necrosis with blood lakes and the kidneys showed atrophic glomeruli, mild interstitial fibrosis, tubular dilatation, and atrophy [117]. Although very few untreated mice lived beyond 6 months of age, surprisingly they did not show lethal hepatic or renal lesions. The significance of this apparently paradoxical finding has yet to be determined [118].

The ***L-G6pc^-^/^-^ mouse model*** is a conditional G6P_C_-liver-deficient model that allows a temporal regulation of *G6pc* deletion in the liver by specific excision of exon 3 in both *G6pc* alleles (using an inducible Cre-lox strategy) when TM is administered, thus avoiding the premature death of the mice and leading to a long-term physiopathology model [128]. As endogenous glucose production, specially from the liver, is critical during the neonatal period, TM was not administered to the mice until they were 6–8 weeks old. Normal survival rates were observed in TM treated mice. Pathophysiological symptoms were similar to humans in most cases, except for growth rate retardation, which was marginally delayed [128]. Hypertriglyceridemia and hyperuricemia were observed after 10 days of TM administration, with three times higher triglyceride and uric acid plasma concentration as well as twice cholesterol concentration compared with control mice. *L-G6pc^-^/^-^* mice also showed a 50% increase in plasma lactic acid concentration compared to WT mice. The aforementioned biochemical alterations in blood remained for 1 month after gene deletion, and returned to normality after 18 months (except for cholesterol) [128]. Hepatomegaly with high glycogen and triglyceride levels and steatosis was also observed after 10 days of TM administration, showing enlarged and pale livers, which increased with time, probably leading to the development of eHCA. However, kidneys and intestines did not have much glycogen content and parameters were normalized after 18 months. HCAs were analyzed by resonance imaging in mice, detecting small liver nodules in 30% of them after 1 year and bigger liver nodules in all mice at 18 months, allowing to study these long-term tumors [128].

The ***K-G6pc^-^/^-^ mouse model*** lacking G6P_C_ subunit specifically in the kidneys was also developed by an inducible Cre-lox strategy [129]. In these mice, Cre recombinase inducible expression was under the control of the kidney androgen-regulated protein (Kap) promoter, which is very active in the proximal tubules of the kidney. As in the case of the *L-G6pc^-^/^-^* model, mice were treated with TM at 6–8 weeks to induce the excision of *G6pc* exon 3 in the kidneys. In contrast to total *G6pc^-^/^-^* mice, growth was not affected as body and liver weight were similar to control mice [129]. Hepatic G6Pase activity was increased by 40% in *K-G6pc^-^/^-^* mice compared with WT mice. Normal life expectancy was observed and blood levels of cholesterol, triglycerides, lactate and urea were normal, although uric acid concentrations were somewhat higher than in WT mice. Affected mice presented an enlargement of kidney tubular epithelial cells, associated to a marked increase in their glycogen stores [129]. Additionally, important alterations in the urinary filtration barrier were also observed, sometimes accompanied by the development of microalbuminuria and electrolyte imbalance [129]. The accumulation of G6P caused the activation of the *de novo* lipogenesis pathway and the subsequent accumulation of triglycerides in the *K-G6pc^−^/^−^* mice kidneys activated the renin-angiotensin system, which finally produced an increased expression of the transforming growth factor β1 (TGFβ1). This resulted in partial epithelial-mesenchymal transition (EMT)-like changes, highlighted by a decrease in epithelial marker expression and an overexpression of the mesenchymal marker fibronectin [129].

The ***I-G6pc^-^/^-^ mouse model*** lacking G6P_C_ protein specifically in the intestine was developed by an inducible Cre-lox strategy similarly to previous *K* and *L-G6pc^−^/^−^* models. Mice expressed the inducible Cre recombinase under the control of the *Villin* gene promoter. As in previous models, mice were treated with TM at 6–8 weeks to induce the excision of *G6pc* exon 3 in the intestine. The specific deletion of G6Pc protein in the intestine was persistent for more than one year but typical symptoms like retarded growth, diarrhea or intestine inflammation were not observed [130]. *I-G6pc^-^/^-^* mice exhibited normal growth rate when fed on a standard rodent starch-enriched diet (SED; 20% energy intake from protein), and did not show a lower food intake or body weight when SED was replaced by a protein enriched diet (65% energy intake from protein). Thus, these mice were no longer sensitive to the satiety effect induced by food proteins [130].

##### Mouse Models for GSD-Ib

The ***Slc37a4^-^/^-^ mouse******model*** was generated disrupting the *Slc37a4* gene by replacing exon 1 and the flanking intron 1 sequence with a neo cassette and inserted into a targeting vector containing a herpes simplex virus thymidine kinase for negative selection [131]. The *Slc37a4^-^/^-^* mice phenotype mimicked the clinical phenotype in patients, as they exhibited a lower post-natal development, hypoglycemic seizures (requiring glucose therapy), as well as hyperlipidemia, and increased uric acid and lactate plasma levels. Mice presented with hepatomegaly and nephromegaly with marked glycogen accumulation in the hepatocytes of the liver and kidney tubular epithelial cells. These mice also exhibited reduced body weight, severe neutropenia and neutrophil dysfunction. As in the case of the *G6pc^-^/^-^* mice, premature death is observed in these animals, although when glucose therapy is implemented within 24 h of birth, 77% of *Slc37a4**^-^/^-^*** mice survive weaning [131].

The ***TM-Slc37a4^-^/^-^ mouse model*** is a conditional G6P_T_-deficient model that allows a temporal regulation of *Slc37a4* gene deletion with the administration of TM, avoiding the premature death of mice and leading to a long-term physiopathology model [135]. *TM-Slc37a4^-^/^-^* mice were generated using an inducible Cre-lox strategy, which resulted in the specific excision of exons 2, 3, 4 and 5 in both *Slc37a4* alleles when TM was administered. TM treatment was administered in mice at 6–8 weeks for 5 consecutive days. Except for the lack of growth retardation, the phenotype was similar to that of patients (i.e., hepatomegaly and nephromegaly with glycogen accumulations in these two tissues, increased serum cholesterol and triglyceride levels, as well as neutropenia and neutrophil dysfunction and mild even monocyte dysfunction) [135].

##### Evaluated Treatments in Animal Models

A complete list of the different treatments evaluated in the different GSD animal models can be found in Table 5.

(a) Gene therapy for GSD-Ia

For the ***Maltese******canine model***, intravenous administration (1.6–7 × 10^11^ pfu/animal) of adeno-associated virus serotype 2 vectors (AAV2) encoding *G6pc* was performed to three affected puppies on days 3 or 4 of life [111]. Fasting glucose levels were normalized for two puppies and a correction of fasting cholesterol, triglycerides and lactic acid levels were also observed for one of these animals after 8 weeks of age. The liver G6Pase activity was significantly increased whereas liver glycogen and lipid content were significantly reduced (with the greatest reduction at 11 weeks following administration) for treated puppies, improving the overall pathological phenotype [111]. In further studies, treatment with the AAV2/8 vector (1013 vp/kg) encoding *G6pc* prolonged the survival of all treated puppies to >11 months, no longer required carbohydrate supplementation following the new-born period and only needed to be fed every 6–10 h, showing normal glucose levels while fasting. Additionally, the liver G6Pase activity was increased to the levels observed in normal dogs, while liver glycogen content was significantly reduced [112]. However, additional studies showed that the expression of the transgene clearly diminished with time [113]. Hence, different vectors were tested, and GSD-Ia dogs were treated with helper dependent adenovirus vectors (HDAd) vectors expressing canine *G6pc*; treated dogs presented prolonged survival (>30 months), were protected from hypoglycemia (22 months), showed a significant increase in liver G6Pase activity and a decrease in liver glycogen content [114]. However, long-term complications such as HCA, cytoplasmatic vacuolation, glomeruloesclerosis and cortical tubule nephrosis with renal failure were observed in these treated dogs [114]. Recently, it has been described that four out of five GSD-Ia dogs that were treated with AAV-G6Pase therapy survived up to 8 years, but presented hepatocellular tumors, suggesting some loss of therapeutic efficacy [115].

For the ***G6pc^-^/^-^ mouse model*** the most critical presentation is life-threatening hypoglycemia. In these mice, glucose therapy clearly improved the 20-day survival rate from <1% to ~60% [119]. However, this therapy is not enough to extend the life of these mice beyond weaning (21 days). Thus, *G6pc^-^/^-^* mice were treated with gene therapy using an adenoviral (Ad) vector carrying murine wild-type *G6pc* gene (Ad-*G6pc*) under the control of the constitutive Rous sarcoma virus (RSV) promoter. Two different approaches were evaluated: (1) a single intravenous Ad-*G6pc* infusion (2 × 10^9^ pfu/animal) in 2 weeks-old mice and (2) neonatal low dose (4 × 10^7^ pfu/animal) Ad-*G6pc* infusion. In the first case, treatment restored 19% of normal hepatic G6Pase activity at days 7–14 post-infusion, improved survival and growth and transiently corrected the metabolic abnormalities manifested by *G6pc^-^/^-^* mice; additionally, only one of the 10 animals that were followed up for 56–70 days post-infusion died prematurely, yielding a 84-day survival rate of 90% [119]. In the second case, all neonatal treated mice survived weaning, their G6Pase activity rose to ~16% of normal levels, but presented frequent hypoglycemic seizures and none of the treated mice lived longer than 14 weeks [120]. In both approaches, the *G6pc* gene was not transduced to kidney and the benefits were not significant because of the rapid loss of the vector-mediated gene [119,120,121].

To achieve long-term correction, different recombinant adeno-associated virus (rAAV) vectors were tested in the *G6pc*^-^/^-^ murine model; in a first approach neonatal infusion of AAV2-*G6pc* failed to improve survival in *G6pc*^-^/^-^ treated mice after weaning due to the delayed kinetics of rAAV-mediated transgene expression [120]. To compensate for this limitation, AAV2-*G6pc* was co-infused with Ad-*G6pc* with the goal of prolonging survival through weaning. All treated mice lived for at least 5 months until sacrificed deliberately [120]. G6Pase activity was improved in liver and kidneys and persisted for 8.5 months (~33% of normal activity), declining to ~23% of normal levels at 12 months post-infusion. Additionally, growth rate improved and biochemical parameters such as blood glucose, cholesterol, triglyceride and uric acid levels were normalized. Although some glycogen accumulation was observed [120], there was no hepatomegaly or nephromegaly. However, as Ad-mediated gene transfer has been associated with inflammation and cellular immune responses and there is pre-existing immunity to the most common AAV2 serotype in over 70% of humans [122], other AAV serotypes were evaluated in order to increase transduction efficiency. Hence, the infusion of AAV1 or AAV8 serotypes carrying the murine *G6pc* gene and the hybrid promoter constituted by the cytomegalovirus enhancer sequence and the chicken β-actin promoter (CMV/CB) was tested in *G6pc*^-/-^ mice. Neonatal mice infused with AAV8-*G6pc* (5 × 10^11^ pfu/animal) via temporal vein survived weaning, but at 4 weeks post-infusion G6Pase activity was only detectable in the liver [122]. The addition of a second dose when the mice reached 1 week of age generated ~20% of normal G6Pase activity in the liver but again almost null activity in the kidneys [122]. On the other hand, when two separate infusions of AAV1-*G6pc* were administered in neonatal (first dose) and 1 week-old (second dose) *G6pc*^-^/^-^ mice, ~10 and 7% of normal G6Pase activity was maintained for 57 weeks in the liver and kidneys, respectively, of the infused mice. Additionally, no premature deaths were observed throughout the study [122]. Although at 2 weeks treated animals manifested hypercholesterolemia, hypertriglyceridemia, hyperuricemia and hypoglycemia, their values were completely normalized by 4 weeks. Hepatomegaly and nephromegaly were markedly improved, glycogen content was clearly reduced in the liver and, although some glycogen accumulation was still observed in kidney tissue, no major histological abnormalities were found [122].

In order to evaluate the effect of using distinct promoter sequences in the efficacy of gene therapy, 2 week-old *G6pc*^-/-^ mice were infused intravenously with AAV8-*G6pc* carrying the canine *G6pc* promoter (instead of the CMV/CB promoter); in these mice, a complete correction of growth retardation and a partial correction of fasting hypoglycemia, significant reduction in liver glycogen content, a normalization of blood cholesterol levels, a restoration of liver G6Pase activity to 25% of normal, and a prolonged survival (up to 7 months) were observed in treated mice [123]. Nonetheless, no significant *G6pc* expression was observed in the kidney. On the other hand, when *G6pc*^-/-^ mice were infused with the AAV8-*G6p*c vector driven by the 5′ flanking sequence (−298 to +128) of the human *G6PC* promoter, hepatic G6Pase activity was recovered to almost normal levels with subsequent occurrence of normoglycemia [112]. However, at 26 weeks, fasting and non-fasting blood glucose levels in the treated animals were lower than those in their WT counterparts, and also presented with increased hepatic glycogen and moderate hepatomegaly [112]. The inclusion of further 5′usptream nucleotides of the human *G6PC* promoter sequence (−2864 to −1) to the AAV8-*G6pc* vector completely normalized G6Pase activity in 2–4 weeks-old *G6pc*^-/-^ mice, restored normal blood glucose levels and normal glycogen and lipid storage in the liver, and prevented the development of fasting hypoglycemia [124]. Recently, codon optimization strategy has also been evaluated and when identical amounts (6 × 10^12^ vp/kg) of human G6Pase constructs (rAAV-*G6PC*) and codon optimized constructs (rAAV-co-*G6PC*) were infused in *G6pc*^-/-^ mice, it was observed that those infused with rAAV-co-G6PC presented more hepatic G6Pase activity than those infused with rAAV-*G6PC* [125]. In fact, the rAAV-co-*G6PC* is at the moment being tested in a phase I/II clinical trial for human GSD-Ia (NCT 03517085). Additionally, in these experiments where different levels of G6Pase activity were recovered, it was concluded that mice restoring less than 2% of normal hepatic G6Pase activity are at risk of developing hepatic tumors [125]. Other human G6Pase constructs tested introduced a Serine to Cysteine substitution in the amino acid position 298 of the human G6P_C_ protein, a key residue that impacts enzyme activity, and these rAAV-hG6PC-S298C vectors were found to be as efficient as rAAV-co-G6PC in directing hepatic G6Pase expression in *G6pc*^-/-^ mice and increases the long-term efficacy for treating GSD-Ia [126,127].

(b) Gene therapy for GSD-Ib

When an adenoviral vector containing the human *SLC37A4* gene sequence (Ad-*SLC37A4*) was infused to *Slc37a4^-^/^-^* mice, these animals showed an effective delivery of the transgene to the liver, bone marrow and spleen, but very few levels in the kidney. Hypoglycemia was restored to normal levels and hypercholesterolemia, hypertriglyceridemia, hyperuricemia and lactic acidemia were decreased. Body weight was partly recovered, hepatomegaly due to glycogen accumulation was reduced and neutropenia was restored to 52% of the counts in WT animals. However, there was some premature death in treated mice [132]. In a different study, when *Slc37a4^-^/^-^* mice were infused with the AAV8-*Slc37a4* vector under the control of the CMV/CB promoter, the *Slc37a4* transgene was primarily delivered to the liver and normalization of hypoglycemia was not acceptable enough to avoid long-term complications as HCA [133]. In another study, the safety and efficacy of liver-targeted AAV8-*Slc37a4* gene-therapy in *Slc37a4*^-/-^ mice directed by human *G6PC* or *SLC37A4* promoter/enhancers, respectively, was evaluated. In the case of the *G6PC* promoter, the same 2.8 kb (−2864 to −1) sequence previously tested for the AAV8-*G6pc* vector was used, while for the *SLC37A4* promoter a minimal 1.62 kb sequence was included in the vectors. When tested, both vectors properly delivered the *Slc37a4* transgene to the liver and corrected the metabolic abnormalities found the in murine GSD-Ib model, although the vector that included the 2.8 kb *G6PC* promoter sequence showed greater efficacy [134].

(c) RNAi approach

This strategy was tested injecting *Gys2* siRNA in *L-G6pc^-^/^-^* mice and caused a great reduction in *Gys2* mRNA levels, that resulted in a reduction of hepatic glycogen accumulation, a decrease in ALT and AST blood levels and displayed similar liver morphology that WT controls [240].

#### 2.3.3. GSD Type II (GSD-II; Pompe Disease)

##### Introduction

GSD-II (OMIM #232300) is an autosomal recessive disorder first described in 1932 by the Dutch pathologist Johannes Cassianus Pompe, and is caused by the absence or deficiency of the acid alpha-1-4-glucosidase (GAA), a lysosomal enzyme that catalyzes the cleavage of the α-1,4 and α-1,6-glycosidic bonds of glycogen [12,13,31]. The GAA enzyme is encoded by the *GAA* gene which in humans is located to chromosome 17q25.2-q25.3. Above 500 pathogenic mutations and polymorphisms have been described in the *GAA* gene, most of them reported in a small population or a single family (being the majority of patients compound heterozygotes) [31,32]. A list of these mutations can be found at http://www.pompecenter.nl. GAA deficiency causes lysosomal glycogen accumulation in several different tissues, but clinical symptoms are mainly caused by cardiac and skeletal muscle involvement [31]. The hallmark of Pompe disease (PD) is the presence of swollen, glycogen-filled lysosomes and the massive accumulation of autophagic debris. Both these characteristics are thought to greatly contribute to the development of muscle weakness [33]. PD incidence ranges from 1 in 14,000 to 300,000 births, although the overall incidence is estimated to be around 1 in 40,000 births [16]. This variation depends on geographic region and ethnicity, with a higher incidence in African-Americans, Southern Chinese and Taiwanese population [16]. Clinically, PD can be classified in different subtypes: the classical infantile onset disease (CIOD), the non-classical infantile onset disease (NCIOD) and the late-onset disease (LOD). The CIOD is caused by complete or almost null GAA activity (<1%), and the first symptoms are already present in the first two months of life, including progressive and severe hypertrophic cardiomyopathy, profound muscle weakness and hypotonia, whilst heart and respiratory failure and death might occur during the first 18 months of life [31,32,34]. Feeding difficulties, poor weight gain, delayed milestones and respiratory difficulties with superimposed infections are also common in these patients [12,13,14,15,16]. A similar clinical presentation in infants but with a milder cardiomyopathy and prolonged survival has been classified as NCIOD [242]. The LOD is characterized by low levels of GAA activity (up to ~20%) and can present in children, adolescence and adults with proximal muscle weakness, motor and respiratory deficit, although cardiomyopathy is normally not present in these patients [16,243]. Long-term complications such as scoliosis and lumbar hyperlordosis may appear, and many patients become wheelchair dependent and require assisted ventilation [31,32].

***Treatment:*** The rationale behind enzyme replacement therapy (ERT) is based on the capacity of cells to secrete and internalize lysosomal enzymes. In PD, ERT consists on bi-weekly intravenous infusions of recombinant human GAA (rhGAA; alglucosidase alpha, Myozyme© (ex-US), and Lumizyme© (US); Genzyme, Cambridge, MA, USA) and is the standard of care for PD since 2006 [35]. ERT successfully prolongs the survival of IOPD patients as well as stabilizes disease progression in LOPD patients by ameliorating the cardiac disease [35], although it barely improves the respiratory phenotype [34,35]. These limitations in the ERT efficacy might be explained by the short rhGAA plasma half-life of, its reduced uptake in skeletal muscle and nervous tissue, as well as its high immunogenicity [36,244]. CNS abnormalities and signs of cognitive decline have been reported in chronic CIOD patients treated with ERT [37]. These limitations have prompted the research to develop more effective second-generation drugs consisting on rhGAA with higher affinity for skeletal muscle cells [38,39,40,41], and search for alternative and/or adjunct therapies such as substrate reduction therapy, inhibition of autophagy and modulation of mTORC1 signaling, chaperone therapy, stimulation of lysosomal exocytosis and antisense oligonucleotides among others [31,32,36,42,43,44].

Gene therapy has also been evaluated in PD patients. Five children with chronic ventilator and severe phrenic neuromuscular dysfunction were enrolled in a phase I/II clinical trial of rAAV1-hGAA intramuscular gene transfer. The results in this trial showed that rAAV1-hGAA was safe and improved respiratory function [45,46,47]. Currently, two successive intramuscular administrations of an AAV9 vector expressing GAA are being evaluated in patients with LOD in a phase 1/2 clinical trial (NCT02240407) [32]. Additionally, two Phase I clinical trials of AAV vector-mediated liver depot gene therapy have also been initiated (NCT03533673 and NCT04093349) [48].

##### Animal Models

(a) Cattle models

Generalized GSD-II was described in ***Shorthorn cattle*** in 1977 by Richards et al. [136]. Tissue homogenates of calves retain some residual GAA activity (about 3% of normal levels) [137]. More than 50% of affected cattle showed generalized muscle weakness, respiratory distress, clinical and pathological signs of congestive heart failure with an increased heart weight. In *Shorthorn cattle*, the mutation in *Gaa* gene is a dinucleotide deletion in exon 18 (c.2454_2455delCA), which is lethal.

In ***Brahman cattle***, GSD-II is caused by a dinucleotide deletion in exon 7 (c.1057_1058delTA) of the *Gaa* gene, resulting in frameshift, and transition of cytosine to thymine in exon 13 (c.1783C>T). These two mutations cause the appearance of two premature termination codons. Other mutations associated to the disease phenotype have been described as well: a transition of cytosine to thymine in exon 9 (c.1351C>T), producing a decrease in GAA activity levels, and a transition of guanine to adenine in exon 16 (c.2223G>A), which results in a silent mutation [138,139]. The incidence of carriers in Australia is 15% [140]. At 6-months of age, the common signs of calves are ill-thrift and muscular weakness. GAA activity was reduced in peripheral blood lymphocytes and skeletal muscle. There are cytoplasmic vacuoles in brain, spinal cord, skeletal muscle, myocardium and Purkinje fibres [141].

(b) Canine model

The possible occurrence of GSD-II in a Lapland dog was first mentioned by Mostafa in 1970 [142], and it was later demonstrated through fibroblasts complementation studies in which human PD cells failed to complement alpha-1-4-glucosidase activity in heterokaryon cells [143]. Clinical presentation included mega-esophagus, exercise intolerance, and recurrent emesis [144]. Glycogen accumulations consisting of membrane bound vacuoles were present in the heart, skeletal muscle, and smooth muscle. The genetic basis has been delineated as a c.2237G>A change corresponding to the p.W746X nonsense mutation in the *Gaa* gene [145]. The fact that the p.W746X mutation has been also reported in human patients and is also found in dogs suggests that the chromosomal region around c.2237G is a mutation hotspot [145].

(c) Mouse models

The ***6^neo^/6^neo^ mouse model*** (also known as *B6;129-Gaa^tm1Rabn/J^*) is the most widely used preclinical PD model. It has a neo cassette inserted within exon 6 of the *Gaa* gene, with loxP sites placed in the disrupted exon 6 flanking introns [146]. These mice have reduced survival (~50% of mortality at 10 months) [147,148], and neither GAA protein nor enzymatic activity was detected in heart, skeletal muscle, brain or tail [146]. Glycogen accumulation was observed at 3 weeks of age, and over the time the lysosomes increased in size and number. At 8 weeks there were PAS-positive inclusions in heart and skeletal muscle and at 14 weeks the diaphragm also showed PAS positive vacuoles. There was also a significant reduction in the number of myofibrils and signs of sarcomere degradation [146]. Furthermore, muscle fibers presented mitochondria with abnormal morphology and decreased oxygen consumption, increased oxidative stress, autophagic block and activation of apoptosis [149]. Electron microscopy (EM) analyses of the skeletal muscle of these mice revealed large areas of autophagic accumulation containing vesicular structures at different stages of a stalled autophagic process: small and large double-membrane autophagosomes with undigested cytosolic material or glycogen particles, multivesicular bodies, multimembrane structures, autofluorescent material, as well other cellular debris [31]. With regard to muscle performance, at 3.5 weeks of age, mice showed early signs of reduced activity and muscle strength, developing progressive muscle weakness; at 8–9 months, mice showed a weak, wadding gait, progressive muscle wasting and some of them a very poor rotarod performance [146,150]. However, no significant alteration was detected in lung function, even at 9 months of age [151]. Interestingly, the PD phenotype in this model was observed to be less severe in females than in males [148].

In order to study the role of autophagy in PD, two different muscle-specific autophagy-deficient models were generated on the *6^neo^/6^neo^* background:

The first autophagy-deficient mouse model, ***AD-6^neo^/6^neo^*** (also known as *AD-GAA-KO or HSAcre:Atg5^F/F^:GAA^-/-^*), contains a Cre recombinase transgene under the control of the human skeletal actin promoter (HSA-Cre) and an *Atg5* gene, in which exon 3 is flanked by loxP sites [183]. The *HSA* promoter drives the expression of Cre recombinase in both fast and slow muscle fibers resulting in muscle-specific inactivation of *Atg5*, a critical gene in autophagosome formation [184]. Suppression of autophagy in muscle of these mice, prevented the autophagic build-up observed in the *6^neo^/6^neo^*, but developed a more severe phenotype, with severe muscle wasting and atrophy, kyphosis, waddling gait and growth retardation. By the age of 6–7 months these mice breathe with difficulty, develop a near paralysis of hind limbs and many begin to die [183]. However, both *6^neo^/6^neo^* and *AD-6^neo^/6^neo^* contain a comparable amounts of glycogen in muscle, raising the question whether macroautophagy in muscle is not the major route of glycogen delivery to the lysosome in adult animals [183].

In order to assure that the *AD-6^neo^/6^neo^* phenotype resulted from the suppression of autophagy alone and not from genetic manipulations or the additional autophagy-independent role of *Atg5*, a second muscle-specific autophagy-deficient mouse model was developed. In this case, the second autophagy-deficient mouse model, ***AD2-6^neo^/6^neo^ mouse model*** (also known as *MLCcre:Atg7^F/F^:GAA^-/-^*), was developed by excising a large segment of the *Atg7* gene in fast muscle fibers by Cre recombinase [185]. As in the case of *AD-6^neo^/6^neo^,* autophagic build-up was not present in these mice. However, in contrast to what occurred in *6^neo^/6^neo^* and *AD-6^neo^/6^neo^* mice, there was a significant reduction in glycogen levels in skeletal muscle fast fibers [185]. It is not clear why these two autophagy-deficient strains have different amounts of glycogen.

Beyond these *6^neo^/6^neo^* mice models, other PD murine models have been developed:

The **Δ*6/*Δ*6 mouse model*** is derived from the *6^neo^/6^neo^* as the *Gaa* exon 6 sequence containing the neo cassette was totally excised from early embryos using Cre-lox strategy, thereby resulting in an in-frame deletion. These mice were similar to the *6^neo^/6^neo^* animals with respect to the levels of enzyme activity, absence of protein and accumulation of lysosomal glycogen in skeletal muscle, heart and diaphragm. However, while the *6^neo^/6^neo^* mice are weak in the open field and show poor performance in wire hang tests, this is not the case for Δ*6/*Δ*6* mice. Besides, Δ*6/*Δ*6* mice did not develop any clinical symptom up to 6.5 months [146], and although longer observations (up to 16 months) showed that clinical symptoms worsened with age, clinical manifestations did not reach the severity of those in *6^neo^/6^neo^* mice [150]. The phenotypic differences between these two models are difficult to explain, although it should be noticed that both strains are of different genetic background since the creation of the Δ*6/*Δ*6* model required mating to a strain bearing the Cre recombinase (FVB/N), while the *6^neo^/6^neo^* mice were bred onto a C57BL/6 background [146].

The ***13^neo^/13^neo^ mouse model*** has a neo cassette inserted within exon 13 of the *Gaa* gene [186], and mice presented a clinical phenotype which was indistinguishable from that of their control littermates until 9 months of age, although at 32 weeks several mice had an abnormal electrocardiogram as well as cardiac enlargement as revealed by radioscopy [186]. GAA activity was reduced by 98% in liver, spleen, kidney, thymus, lung, heart, triceps, femoral and sural muscles, tongue, cerebellum and brain. In the intestine, the enzyme activity was reduced by 25%. The low activity of skeletal muscle and heart correlated with those observed in patients. At 8 days after birth, although the femoral muscle was unaffected, it showed lysosomal accumulation of glycogen, and the heart also contained small glycogen lysosomes, as well as liver, smooth muscle cells of blood vessels, Schwann cells and anterior horn cells. At 6 weeks, glycogen vacuoles were more abundant forming large vacuoles and showing continuity along the myofibrils at 13weeks and skeletal muscles were positive for acid-phosphatase staining [186].

In the **Δ*14^neo^/*Δ*14^neo^ mouse model,*** exon 14 of the *Gaa* gene and its flanking intronic sequences (1.3 kb) are replaced by a neo cassette, resulting in a frame shift (Pro629/shift) and premature termination 36 amino acids downstream [150]. Neither *Gaa* expression nor activity was detectable in tail skin, muscle heart and brain. PAS-positive diastase sensitive material in vacuoles was observed in heart, skeletal muscle and brain [150]. At 4 months of age, Δ*14^neo^/*Δ*14^neo^* showed signs of reduced activity in an open field environment, thereby closely matching the results obtained for the *6^neo^/6^neo^* mice. In fact, the clinical course of the disease in the Δ*14^neo^/*Δ*14^neo^* mice was indistinguishable from that of the *6^neo^/6^neo^* mice [150].

The ***Gaa KO^DBA^ mouse model*** was obtained by crossing the *6^neo^/6^neo^* mice with DBA2/J mice, resulting in a *Gaa* mouse strain homologous for *Ltpb4*^Δ36^ allele (Latent TGF-β binding protein 4) [151]. This mouse model was generated in order to exploit the DBA2/J genetic background, and in particular the *Ltpb4* modifier gene, which has been described to act as a modulator of the severity of dystrophies in mice and humans [151]. *Gaa KO^DBA^ mice* showed reduced survival (~50% of mortality at 6 months), pathological glycogen storage in muscle and nervous system, reduced muscle strength and their respiratory function was significantly affected [151]. Additionally, these mice presented with an impairment of the skeletal muscle autophagy flux, cardiac hypertrophy and poor rotarod performance (with the latter suggesting coordination defects) [151]. As in the case of the *6^neo^/6^neo^* mice, the disease phenotype of the *Gaa KO^DBA^ mice* was less severe in females than in males [151].

Finally, the ***Gaa^c.1826dupA^ mouse model*** was generated using the novel CRISPR-Cas9 homology-directed recombination to harbor the orthologous *Gaa* mutation c.1826dupA (p.Y609*), which causes human IOPD [245]. These mice showed a major decrease in GAA activity (>95% in heart, diaphragm and gastrocnemius), massive increases in glycogen content (185 and 28 fold increases in the heart and gastrocnemius, respectively), autophagy impairment, decreased forelimb muscle strength, significant reduction in body mass and abnormal cardiac function [245].

In addition to these models, ***transgenic mice that constitutively expressed human GAA cDNA*** under specific muscle or liver promoter were developed from *6^neo^/6^neo^* mice in order to study the efficacy of skeletal muscle and liver as locations for gene replacement therapy [246]. In this regard it was observed that when the transgene was constitutively expressed in the entire muscle mass, no secretion by muscle cells and no uptake by distant tissues (such as liver, heart or diaphragm) occurred. The liver, however, proved to be a suitable organ for production and secretion of GAA protein into the bloodstream and uptake in peripheral tissues [246].

(d) Quail models

A natural animal model of PD in Japanese quails was described in 1983 [187]. Affected quails showed progressive muscle weakness, were unable to move their wings (which were fixed alongside of the body) and could not right themselves when placed on their backs or sides [187]. The *pectoralis* muscle was the most severely affected muscle, presenting an edematous and pale aspect and being partially replaced by fat tissue. Muscle fibers showed vacuoles of different sizes as well as glycogen depots [187,188]. Four weeks after hatching enzymatic activity was decreased in muscle to less than 10% of normal values [188]. With age, muscle degeneration/regeneration was evident and skeletal muscle tissue was replaced by fatty tissue [189]. Quails did not develop cardiomyopathy although glycogen accumulated in the myocardium [189]. The disease was caused by a defect in the maturation and processing of the GAA protein, as the precursor (~110 kDa) protein was present in the affected quails, but not its mature form (~98 kDa) [190]. Although the usefulness of this model has become outdated, quails deserve a special mention because they were the first to be used for testing ERT [189].

##### Evaluated Treatments in Animal Models

(a) ERT

As indicated above, quails were the first animal model in which ERT was tested [189]. Quails treated with rhGAA improved muscle performance and strength, increased GAA activity in the skeletal muscle, liver, heart, spleen, kidney, lung, and testis, reduced the glycogen content in all tissues analyzed, and more importantly, to almost normal levels in liver and heart [189]. Due to this positive results, ERT was further investigated in the *6^neo^/6^neo^* mice in several different approaches including the introduction of additional mannose-6-phosphate moieties onto rhGAA to enhance its uptake by skeletal muscle cells [152], the co-administration, improving its lysosomal delivery by fusing it with glycosylation-independent lysosomal targeting tag [40], diminish the immunogenicity response using phosphatidylinositol (PI) containing liposomes as vehicles [153], or the co-administration with anti-B-cell activating factor drugs [154] among others.

(b) Gene therapy

Several different gene therapy approaches have been evaluated for GSD-II using the *6^neo^/6^neo^* mice model as the main target. Different vectors, promoter sequences, doses and infusions strategies have been tested in order to minimize the disease phenotype found in these mice. Intramuscular injections of rAAV2-*Gaa* (1 × 10^9^ i.u) resulted in near normal GAA activities and reduced glycogen accumulation for at least 12 weeks as well as preserved skeletal muscle contractile force in mutant mice, while intramyocardial injections also recovered GAA activity for at least 6 weeks [155]. In a different study, the AAV2/6 vector containing the muscle creatine kinase (MCK) promoter/enhancer expressed human *GAA* at approximately 100-fold above the normal levels 6 weeks after the injection (1 × 10^11^ particles) in the gastrocnemius of the *6^neo^/6^neo^* mice, significantly reducing the glycogen content in the muscle; additionally, this high degree of transgene expression exceeded the threshold for GAA protein secretion from muscle by approximately 8-fold [156]. Gel mediated delivery of the rAAV2/1-*GAA* (1 × 10^11^ vg) in the diaphragm of the *6^neo^/6^neo^* mice resulted in almost normal GAA activity levels, a reduction in the amount of stored glycogen and a significant improvement in diaphragm contractility, even up to 9 months post-treatment [157]. As tongue weakness is prevalent in PD, and the hypoglossal-tongue motor system provides an ideal experimental model to evaluate the retrograde gene delivery to motorneurons (i.e., muscle to motorneuron), a single intralingual injection of AAV1-*GAA* or AAV9*-GAA* (1 × 10^11^ vg) was performed in the *6^neo^/6^neo^* mice and resulted in persistent transgene expression both in lingual myofibers and brainstem XII motorneurons [158]. All of these assays improved the characteristics of the disease in mice, however, the effect of treatment was confined to AAV-injected muscle, due to the local nature of intramuscular gene transfer and to the low enzyme cross-correction achieved. To solve this problem, different approaches were tested. For muscle targeted systemic gene transfer, various studies using different AAV and different muscle promoters have been performed [156,159,160,161,162,163,164] in *6^neo^/6^neo^* mice leading to similar findings of improvement of the respiratory phenotype, muscle strength and morphology, although there was not a complete glycogen correction and not in all studies [165]. For liver targeted systemic transfer various studies using different AAV and liver-specific promoters have been tried too in mice models, showing an efficient liver transduction with 10 to 100 times lower AAV vector doses needed [147,148,152,166,167,168,169,170,171,172]. In this case, the effects are mainly based on cross-correction, as GAA enzyme is only produced by hepatocytes, secreted to the circulation and taken up by target cells, showing a glycogen clearance in the heart and diaphragm but not as much in the skeletal muscle probably due to an insufficient native secretion of GAA. An AAV vector encoding GAA under the control of a tandem liver/muscle promoter was administered in *6^neo^/6^neo^* mice and resulted in persistent therapeutic efficacy with circulating enzyme levels that allowed the cross-correction of several target tissues, including CNS [173].

The last approach is the administration of different AAV-GAA targeting the central nervous system (CNS) by intracerebroventricular delivery resulting in glycogen reduction only in CNS but not in muscle [174] and intrathecal administration resulting in glycogen reduction in CNS and in heart [175].

(c) Other treaments

Besides ERT and gene therapy, several other treatments have been tested in GSD-II animal models. These include pharmacological chaperones [176,177], transplantation of lentiviral vector *GAA*-modified hematopoietic stem cells (HSCs) [178,179,180], leucine supplementation [181] and satellite cell activation [182].

#### 2.3.4. GSD Type III (GSD-III; Cori Disease)

##### Introduction

GSD-III (OMIM #232400) is an autosomal recessive disorder with an estimated incidence of 1:100,000 live births [49]. It is caused by pathogenic mutations in both alleles of the *AGL* gene which encodes the glycogen debranching enzyme (GDE). GDE is a dual-function protein that has two different enzymatic activities: 1,4-a-D glucan:1,4-a-D-glucan 4-a-D-glycosyltransferase (EC.2.4.1.25) and amylo-1,6-glucosidase (EC 3.2.1.33) [50,51]. Both glycogen phosphorylase and GDE cooperatively degrade cytoplasmic glycogen into glucose. A deficiency in either of the two GDE enzyme activities produces, in the affected tissues, an accumulation of glycogen with short outer chains, known as phosphorylase limit dextrin (PLD) [49]. In patients with GSD-III, the liver and the muscle are the main tissues affected, as these are the two tissues with the highest total capacity for glycogen storage [52,53]. In fact, the majority of patients (>80%) do not present enzymatic activity neither in liver nor in muscle (GSD-IIIa); a few (<15%) have only the liver affected (GSD-IIIb), and in very rare cases the affectation only occurs in the glucosidase activity (GSD-IIIc) or in the transferase activity (GSD-IIId) [54,55]. Clinically, the patients usually present with hepatomegaly, fasting hypoglycemia, hyperlipidemia, growth retardation and variable myopathy and cardiomyopathy. The main clinical manifestations during childhood are hepatomegaly that appears in the first weeks of life, hypoglycemia, hyperlipidemia, and growth delay [56]. These manifestations tend to be slowly attenuated during adolescence, although cirrhosis and hepatic adenomas ultimately leading to liver transplantation have also been reported in some patients [57,58,59,60]. There is a high prevalence of myopathy among adult patients with GSD-III (70%), starting from the 3rd or 4th decade of life [61], which manifests as weakness in the trunk and proximal and distal muscles, a reduced capacity to jump or walk for a long time and decreased handgrip-strength. Another characteristic of this disease is that not all muscles are equally affected, with some muscles being more affected than others [55]. Regarding the histology, the samples show the presence of non-membrane bound glycogen vacuoles in the subsarcolemmal region and deep inside muscle fibers, which ultimately disrupt and displace the rest of cell constituents. In addition to the skeletal muscle, cardiac involvement in this disease has been known since 1968, when the existence of significant glycogen depots in cardiac muscle were first reported [62]. As of today, left ventricular hypertrophy is present in at least 50% of affected patients, which may evolve to severe cardiomyopathy [63,64]. In concordance with the affected tissues, higher-than normal levels of liver transaminases (alanine transaminase, aspartate transaminase, alkaline phosphatase) or of the muscle damage indicator creatine kinase (CK) are prevalent among patients with GSD-III. Other manifestations reported in patients with GSD-III include osteoporosis and polycystic ovaries [65].

***Treatment:*** Current treatments for GSD-III are mainly symptomatic and are not curative. The most frequently used therapies are dietary such as providing uncooked corn starch to prevent hypoglycemia at young ages, and high-protein diets, which have been shown to reverse the extent of cardiomyopathy associated with GSD-III [64,247,248]. In addition, the use of medium-chain triglycerides has shown positive therapeutic effects in patients with GSD-Ia [249] and GSD-IIIa [250]. However, dietary therapies do not prevent the long term complications of GSD-IIIa, including hepatic cirrhosis, HCA, hepatocarcinoma (HCC), cardiomyopathy, and myopathy [57].

##### Animal Models

(a) Canine model

The naturally occurring curly coated retriever (CCR) dog model of GSD-IIIa is caused by a homozygous frameshift mutation in exon 32 of the *Agl* gene that leads to the deletion of 126 amino acids at the C-terminus of the GDE protein. This model was described for the first time by Gregory and collaborators and did not show GDE enzymatic activity either in the liver or in the muscle [191]. Liver biopsies showed large accumulations of glycogen, but neither inflammation nor fibrosis was observed. However, subsequent studies analyzed the progression of the disease phenotype in this model at different ages (4, 12, 16, 19, 20 and 32 months as well as 11 and 12 years) [192,193]. These studies revealed a progressive enlargement of the liver, with large nodules and cirrhosis found in the oldest dogs. Hepatic glycogen content was reported to be much higher in CCR dogs than in healthy (WT) dogs and progressively increased reaching its peak at 12 months (6-fold higher than WT values), then beginning to decrease until 32 months (2.5-fold higher) [192,193]. Thus, in less than two years the liver lost more than half of its glycogen content, probably due to the replacement of liver tissue by fibrous tissue [192]. In fact, hepatic fibrosis leading to cirrhosis was found in one dog (32 months-old) [193]. Similarly, a gradual increase in skeletal muscle glycogen was observed until 16 months of age (7 fold higher than WT values), thereafter decreasing until 32 months of age (5-fold higher) [192,193]. Thus, there was a progressive muscle damage caused by a gradual increase in glycogen levels which finally resulted in complete disruption and displacement of the contractile apparatus with the entire sarcoplasm occupied by glycogen [192]. Interestingly, some glycogen deposition was also observed in the extraocular muscles in one of the dogs (32 months-old) [193]. Cytoplasmic/sarcoplasmic glycogen deposits were also observed within cardiomyocytes, Schwann cells from sciatic and phrenic nerves and extraocular muscles [193]. Serum biochemistry showed high levels of liver transaminases, slightly elevated CK levels, with the rest of routine variables within normal limits [192].

(b) Mouse models

The ***Agl_EX5_^-/-^ mouse model*** consists of a constitutive *Agl-*KO mouse developed by inserting the mouse *engrailed 2* (*en2*) gene splice acceptor and a poly (A) tail cassette between exons 5 and 6 of the mouse *Agl* gene. Thus, after splicing the *Agl* mRNA does not contain exons downstream of exon 5, resulting in a C-terminal deletion of GDE lacking the two catalytic domains [49]. As a consequence, this model shows undetectable levels of GDE protein associated with a clear reduction in enzyme activity levels in liver, muscle and heart (~11% of normal in liver and muscle, respectively, and ~22% in heart) [49]. Although ***Agl_EX5_^-/-^*** mice exhibited normal appearance, body weight and life-span, when mice were subjected to repeated fasting episodes, 50% of them died prematurely before 50 weeks of age. Hepatomegaly with enlarged hepatocytes presenting pale cytoplasm was already present in 4-weeks-old mice, and an expanded area of fibrosis was observed in 28-weeks-old mice. Severe glycogen accumulation was observed in liver, heart, diaphragm, as well as in quadriceps and gastrocnemius muscles [49].

The ***Agl_EX32_^-/-^ mouse model*** consists of a constitutive *Agl*-KO mouse developed by deleting the genomic region spanning exons 32 to 34 of the *Agl* gene which encoded the last 114 amino acids of the GDE protein comprising the glucosidase and glycogen binding domains [194]. This model showed a complete absence of GDE protein and enzyme activity as well as high glycogen depots in the liver and muscle, thereby causing hepatomegaly and muscle impairment accompanied by a high mortality rate within the first year of age [194]. In the liver, glycogen levels were 2.6 fold-higher than in their WT counterparts, presenting an abnormal structure (phosphorylase dextrin limit). Although liver parenchyma was darker and hepatocytes cells were enlarged, no signs of inflammation, fibrosis, cirrhosis or adenomas were observed at any age [194]. In the skeletal muscle, glycogen levels (located at the subsarcolemmal and intrasarcoplasmic space) were 22 fold-higher than in WT mice, and showed an abnormal structure (PLD). In fact, a large proportion of the sarcoplasm was occupied by glycogen, causing significant changes in the muscle fiber architecture [194]. Consequently, ***Agl_EX32_^-/-^*** mice had poor exercise performance as determined by treadmill, rotarod and hind-print test. Regarding serum biochemistry, these mice presented lower basal and fasting glucose levels as well higher liver transaminases and CK levels than WT mice. However, hyperlipidemia, which is frequent in patients, was not observed in this mouse model [194].

*The **Agl_EX6-10_^-/-^ mouse model*** consists of a constitutive *Agl*-KO mouse developed by replacing exons 6–10 of the *Agl* gene with a neomycin-expressing cassette [195]. These mice showed a complete absence of GDE protein and activity in the liver and skeletal muscle and presented glycogen accumulation in liver, heart, skeletal muscle and brain. Additionally, 6 month-old *Agl_EX6-10_^-/-^* mice presented hypoglycemia and hepatomegaly [195]. Survival (monitored up to 18 months) was not impaired but in terms of exercise these mice presented a significant decrease in muscle strength, although no differences were observed in rotarod performance and in total distance travelled [195].

Interestingly, another Agl-KO mouse developed by deletion of exons 6-10 of the *Agl* gene has been recently generated by Lim and collaborators (***Agl_EX6-10_^-/-^2****)* [196]. These mice did not present GDE protein in the liver, heart and skeletal muscle, and high glycogen accumulation was observed in the liver, heart, skeletal muscle and smooth muscle, and to a minor degree in some regions of the brain and spinal cord [196]. Additionally, these mice also presented hepatomegaly, progressive liver fibrosis, exercise intolerance and impaired muscle functions. However, in contrast to *Agl_EX6-10_^-/-^* mouse model, *Agl_EX6-10_^-/-^2* mice did not show hypoglycemia **[196]**.

##### Evaluated Treatments in Animal Models

In a *Agl^-/-^* mouse model (whether this model is the *Agl_EX5_^-/-^, Agl_EX32_* ^-/-^, or a different one is not specified in the report) it was demonstrated that the inhibition of *Gys2* expression by means of RNAi techniques caused a clear decrease in the synthesis of glycogen, a reduction in the levels of glycogen in the liver, as well as an evident decrease in hepatomegaly, fibrosis and nodule development in these mice, indicating that GSD-III liver phenotype of *Agl^-/-^* mice is highly reversible with GS-L reduction [240].

In the ***Agl_EX6-10_^-/-^ mouse model*** gene therapy was evaluated using different strategies; first, mice treated with an AAV-GAA vector (at 1 × 10^11^ or 1 × 10^12^ vg/mouse) presented a significant decrease in glycogen levels in the liver but not in the skeletal muscle. Additionally, this treatment did not lead to a significant improvement in blood glucose or hepatomegaly [195]. *Agl_EX6-10_* mice were then treated with dual overlapping AAV vectors expressing the *Agl* transgene. In treated mice, GDE protein was detected in the heart and skeletal muscle, but not in the liver. To solve this problem, the dual vector approach was tested under the transcriptional control of a liver-specific promoter. In these experiments, treated mice presented GDE protein in the liver, a clear reduction in liver glycogen content accompanied by an increase in blood glucose levels, although hepatomegaly was not corrected [195]. In the ***Agl_EX6-10_^-/-^2 mouse model*** gene therapy was also evaluated using an AAV vector expressing Pullulanase, a small bacteria GDE (from *Bacillus subtilis*). This treatment significantly reduced glycogen accumulation in the liver and skeletal muscle and clearly improved liver and muscle functions [196].

#### 2.3.5. GSD Type IV (GSD-IV; Andersen Disease)

##### Introduction

GSD-IV (OMIM #232500) is a rare autosomal recessive disorder (less than 1% of all GSDs patients) [16], caused by pathogenic mutations in *Gbe1* gene (located to chromosome 3p12), which encodes the glycogen branching enzyme (GBE), [16,23,66]. As GBE activity is lost, glycogen cannot be subjected to the branching process and thus shows an abnormal structure (amylopectin-like) in the liver and skeletal muscle biopsies, with fewer branch points than normal. These abnormal glycogen depots are deeply PAS-positive bodies also known as ‘polyglucosan bodies’ [16].

Clinical manifestations are quite variable [16,66]. Several mutations have been reported in the *Gbe1* gene, leading to different amounts of residual enzyme and to classic or non-classic forms of the disease, with different degrees of affection of skeletal muscle, cardiac muscle, liver and nerves, as well as different ages of symptom onset [66]. The classic form appears in infancy, usually at 18 months, with altered growth, portal hypertension, hepatosplenomegaly and progressive liver cirrhosis leading to death by 3 to 5 years of age. There might also be CNS involvement [16,66], as well as adult forms, which present with progressive myopathy resembling muscular dystrophy (i.e., walking difficulties and proximal limb weakness) [16,66]. Even though the maintenance of normoglycemia with diet might help to improve symptoms, the majority of patients will die in early childhood if they do not undergo liver transplantation [23].

##### Animal Models

(a) Horse model

An equine natural model was described in seven *American Quarter Horse foals*. Clinical signs and symptoms were diverse including stillbirth, transient flexural limb deformities, seizures and respiratory or cardiac failure to persistent recumbency. Disease was only evaluated with GBE enzyme activity and quantity of protein assessment, showing in both cases a reduced amount and activity of the enzyme in skeletal muscle and liver. Furthermore, in the oldest foals, abnormal PAS-positive accumulations were found in skeletal muscle, liver and CNS, as it occurs in patients [197].

(b) Cat model

A natural occurring orthologue of human GSD-IV was described in Norwegian forest cats. In these cats a *Gbe1* gene mutation consisting of a 334 bp insertion and 6.2 kbp deletion that extends from exon 11 to exon 12 was reported [198]; this mutation caused an alteration of *Gbe1* mRNA splicing with the subsequent decrease of GBE protein levels in liver and muscle. Phenotypically, most affected cats, as also happens with patients, died the first days after birth, due to the inability of maintaining glucose homeostasis in vital organs. Those cats that survived the postnatal period showed first a reduced voluntary movement capacity, but when they were supplemented with glucose, they appeared clinically normal until the onset of progressive neurological decline, with generalized muscle tremors leading to progressive skeletal muscle atrophy. The large accumulations of glycogen present in neurons and skeletal muscle, led to the analysis of glycogen branching activity in skeletal muscle, which was practically null [198].

(c) Mouse models

The ***Gbe1^neo^/^neo^ mouse model*** was obtained by inserting flippase recognition target (FRT) recombination sites upstream and downstream of *Gbe1* exon 7 sequence and a phosphoglycerate kinase-neo cassette (PGK-Neo) within intron 7 sequence, leading to a reduced synthesis of GBE protein [199]. The reduced expression of the *Gbe1* gene leads to a hypomorphic allele with residual enzyme activity and later onset of disease. Therefore, mice carrying this mutation (*Gbe1^neo^/^neo^*) present a clinical phenotype similar to that of patients with the juvenile/adult onset form of GSD-IV, with widespread accumulation of polyglucosan bodies mainly in brain and skeletal muscle compared with WT. Additionally, these mice respond normally to fasting episodes and glucose bolus, suggesting that storage of glucose as glycogen or polyglucosan bodies can occur very efficiently, even with low GBE activity. However, there are tissue-specific differences in the ability to degrade polyglucosan bodies, with only the liver being able to degrade these structures. No *Gbe1^neo^/^neo^* mice survived beyond 39 weeks [199].

The ***Gbe1^-^/^-^ mouse model*** was obtained by crossing *Gbe1 ^+^/^neo^* mice with FLPe-expressing mouse strain (GT(ROSA)^26Sor-Flpe^) in order to delete the sequences between two FRT sites, (i.e., FLPe-mediated homozygous deletion of exon 7 that completely eliminated GBE activity (*Gbe1^-^/^-^*)). This *Gbe1^-^/^-^* mouse model presented with lethal early onset GSD-IV phenotype, with in utero accumulation of polyglucosan bodies in the liver and skeletal muscle accompanied by a decrease in glycogen in all tissues [199].

The ***Gbe1^ys^/^ys^ mouse model*** was obtained by homologous recombination to knock-in the most common *Gbe1* mutation p.Y329S i.e., c.986A>C (rs80338671) found in adult onset Ashkenazi Jewish descent patients into exon 7 and introducing FRT recombination sequences upstream and downstream of the exon 7, leading to an hypomorphic allele with residual enzyme activity and to a later onset disease. Thus, *Gbe1^ys^/^ys^* mice exhibit a phenotype similar to the human adult onset with late accumulation of polyglucosan bodies in CNS, skeletal muscle, heart and liver, and showing progressive neuromuscular dysfunction and premature death (i.e., before the age of 20 months) [200].

##### Evaluated Treatments in Animal Models

***Gene therapy*** was evaluated by intravenous infusion of a AAV9 vector containing a human GBE expression cassette (AAV-GBE) into 14-days-old *Gbe1^ys/ys^* mice. In AAV-treated mice, GBE enzyme activity highly increased in heart at the age of 3 months, which was consistent with the high copy number of viral vector genome that was detected. GBE activity was also increased in skeletal muscle and brain but not in liver. The glycogen content was normal in skeletal muscle but significantly reduced in liver and brain. A long-term effect was evaluated with AAV-treated mice at 9 months of age, showing a high GBE activity only in the heart tissue and low glycogen levels in liver, brain and skeletal muscle. Overall, AAV treatment resulted in a decrease of alanine transaminase, aspartate transaminase and CK plasma levels and an increase in fasting plasma glucose concentration, showing a long-term benefit of the therapy [201].

#### 2.3.6. GSD Type V (GSD-V; McArdle Disease)

##### Introduction

GSD-V (OMIM #232600) is an autosomal recessive disorder of muscle glycogen metabolism first described by Brian McArdle in 1951 and thus commonly known as ‘McArdle disease’ [67]. It is caused by deficiency of the skeletal muscle isoform of glycogen phosphorylase enzyme (also termed myophosphorylase), which is encoded by the *PYGM* gene located to chromosome 11q13 [68], without affection of the liver (*PYGL*) or brain (*PYGB*) enzyme isoforms, leading to a myopathy [69]. To date, 206 mutations have been described in the *PYGM* gene [70], with the most prevalent mutation in the Caucasian population (e.g., allele frequency of ~55% among Spanish patients, being a nonsense mutation (p.R50X) located in exon 1 of the *PYGM* gene [71]. Myophosphorylase initiates glycogenolysis in skeletal muscle fibers, degrading muscle glycogen by removing (1,4)-alfa-glucosyl units from the branches and releasing glucose-1-phosphate; thus, patients are unable to obtain energy from muscle glycogen stores. This in turn leads to accumulation of subsarcolemmal vacuoles of glycogen [72,73].

The clinical phenotype is usually moderate and appears during childhood or early adulthood, but can sometimes be severe, with disease manifestations starting at a very early age (neonatal) [72,73]. The clinical manifestation typically consists of exercise intolerance, usually in the form of ‘acute crises’ of muscle pain and contractures early during exertion, which can be accompanied by rhabdomyolysis (in ~50% of cases), as reflected by elevated serum levels of CK and sometimes myoglobinuria (as reflected by the presence of ‘dark urine’), which could eventually cause acute renal failure in the more severe cases [72,73]. Exercise intolerance can be triggered by static or isometric exercises (e.g., carrying weights) or dynamic exercise (e.g., running). In addition, patients exhibit a unique feature, the second wind’ phenomenon—that is, a marked improvement in the tolerance to dynamic exercise (e.g., bicycling at a constant, submaximal wattage or walking at a fast pace) after 6–10 min of exertion, with subsequent attenuation of previous tachycardia and muscle pain [72,73]. This phenomenon is due to an increased availability of bloodborne fatty acids and glucose to working muscles (through exercise-induced vasodilation and increased blood flow) after the first minutes of exercise [72,73].

***Treatment:*** No major beneficial effects have been reported in McArdle patients receiving branched chain amino acids [74], depot glucagon [75], dantrolene sodium [76], verapamil [77], vitamin B6 [78] (except for a recent case report [79]), high-dose oral ribose [80], triheptanoin [81], sodium valproate [82] or creatinine [200,201]. On the other hand, because the blockade of glycolytic flux in skeletal muscle fibers occurs upstream the uptake of blood glucose from these cells, pre-exercise ingestion of carbohydrates provides an alternative fuel for working muscles and can alleviate exercise intolerance, especially at the start of exercise [85].

##### Animal Models

(a) Bovine model

The first spontaneous model described for GSD-V was a Charolais cattle, which presented with exercise intolerance and rhabdomyolysis, alteration of electrolyte concentrations in blood, high serum CK, and myoglobinuria [202]. To analyze the cause of the disease in this breed of cattle, *Pygm* cDNA was sequenced and cloned, revealing a point mutation causing a C to T substitution in codon 489, leading to an arginine to tryptophan substitution in the myophosphorylase protein [203].

(b) Ovine model

A second spontaneous animal model for GSD-V was found in a flock of Merino sheep. Animals presented with exercise intolerance and muscle biopsies showed a lack of muscle glycogen phosphorylase and excess of glycogen. Genetic analyses showed an adenine-to-guanine substitution at intron 19 acceptor splice site of the *Pygm* gene causing a deletion of 8 bp with the subsequent disruption of the reading frame and the appearance of a premature stop codon, resulting in removal of the last 31 amino acids from the myophosphorylase protein [204].

(c) Mouse model

The ***Pygm^R50X/R50X^* mouse model** carrying the p.R50X mutation in exon 1 of the *Pygm gene* was generated in 2012 by our group [208]. These mice recapitulate most phenotype traits shown by patients, that is, absence of both myophosphorylase protein content and activity in skeletal muscles, high muscle glycogen content, hyper-CK-emia, myoglobinuria, as well as exercise intolerance (as evidenced by very low performance in wire grip and treadmill tests) [208,209]. Additionally, single muscle contraction studies revealed that contractile force was more reduced in a muscle with a predominantly fast-twitch (i.e., mostly containing myosin heavy chain (MCH) II) fiber phenotype, the *extensor digitorum longus* (EDL), than in one with a slower twitch (MCH I) phenotype, the *soleus* muscle – although an increase in fatigability compared to normal values was also found in the former [210]. From the distinct muscles analyzed in the *Pygm^R50X/R50X^* mice, it appears that the quadriceps and *soleus* are less histologically affected by disease progression than *gastrocnemius*, *tibialis* anterior or EDL muscles [211]. The different muscle fiber types are also distinctly affected by the disease as fiber degeneration caused by massive glycogen stores was principally observed in fibers containing MHC type IIA, IIX and a mix of I/IIA and IIA/IIX, with a lesser degree of degeneration found in pure type I slow oxidative fibers, or in those containing a mix of MHC IIX/IIB or pure MHC IIB fibers [209,210,211]. On the other hand, no major change in fiber type composition was observed in *Pygm^R50X/R50X^* mice in any of the muscles analyzed [211]. Blood analyses showed a decrease in glucose and lactate levels and an increase in ammonia levels with respect to WT mice [211], and increased levels of 4-hydroxynonenal-modified-proteins (a marker of oxidative stress) were observed in the quadriceps of *Pygm^R50X/R50X^* mice [212]. Finally, high levels of perinatal and post-weaning mortality have been observed in the *Pygm^R50X/R50X^* mouse model [211].

(d) Zebrafish model

In this model, the two forms of muscle glycogen phosphorylase encoded by *Pygma* and *Pygmb* genes (which share more than 80% of amino acid sequence identity with human *PYGM*) were knockdown using morpholino strategy and resulted in reduced protein levels in zebrafish morphants, which exhibited altered, disintegrated muscle structure and accumulation of glycogen granules in the subsarcolemmal region [215].

##### Evaluated Treatments in the Animal Models

(a) Compounds inducing re-expression of the (fetal) *Pygb*/*Pygl* isoforms

Several drugs have been evaluated in vivo or in vitro in animal models of McArdle disease based on the upregulation of *Pygb* and/or *Pygl*, which are expressed in the skeletal muscle from embryos but not in mature skeletal muscle. In the case of the ovine model, notexin and sodium valproate have been evaluated [205,206]; when notexin, a myotoxic venom from the Tiger Snake, was injected into the skeletal muscles of the ovine model, it caused skeletal muscle damage and inflammation followed by activation of regeneration processes, which in turn induced the expression of non-muscle *Pygb/Pygl* isoforms. Treatment also induced a reduction in muscle glycogen storage as well as an increase in muscle contraction force [205]. Further investigation on systemic delivery and maintenance of the re-expression is needed. Intramuscular and enteral **sodium valproate (VPA)** administration, a histone deacetylase inhibitor, induced expression of *Pygb* gene in skeletal muscle, thereby causing an increase in the number of glycogen phosphorylase-positive fibers in the treated animals [206]. However, glycogen accumulation was not analyzed and physical activity was not significantly improved, as well as biochemical parameters (CK and aspartate transaminase) [206]. In skeletal muscle cultures derived from *Pygm^R50X/R50X^* mice, VPA induced a dose-dependent increase in *Pygb* expression accompanied by a reduction in intracellular glycogen stores [213]; however, when VPA was administered to *Pygm^R50X/R50X^* mice, no significant changes in skeletal muscle glycogen content and *Pygb* expression were observed (unpublished data).

(b) Gene therapy

Gene therapy could be an excellent therapy for McArdle disease since there is evidence that even residual amounts of enzyme might suffice to attenuate disease symptoms [251]. Gene therapy was tested in vivo for the first time in the ovine model [207]. Treatment was performed in sheep aged between 2 days and 14 months via intramuscular injections of adenovirus 5 (AdV5) and adeno-associated virus serotype 2 (AAV2) containing either the LacZ reporter (control) or human *PYGM* cDNA (intervention) into the semitendinosus muscle of sheep. Myophosphorylase expression was observed in the site of injection and the protein was functional, as indicated by the positive association between the amount of myophosphorylase-positive fibers and the reduction of muscle glycogen content [207]. Despite such good results, expression of *PYGM* appeared to decrease with time, possibly due to an immune response against the human enzyme and also to the specific viral vectors [207]. Gene therapy was also tested in *Pygm^R50X/R50X^* mice via intraperitoneal injections of rAAV8-*Pygm* in the first postnatal days, resulting in *Pygm* expression at 8 weeks of age, as well as in improved skeletal muscle architecture, reduced accumulation of muscle glycogen, and restoration of voluntary running wheel activity to normal levels [214].

(c) Read-through compounds

The most prevalent pathogenic mutations in patients with McArdle disease are premature termination codons (PTC). As such, read-through agents (RTA), which are able to induce the ribosome to bypass a PTC [252,253], are potential therapeutic candidates for this disease. Different RTAs were tested in different McArdle disease cell cultures models: (1) transiently transfected cells with p.R50X plasmid constructs, (2) cells stably expressing p.R50X plasmid constructs and (3) skeletal muscle cells derived from the *Pygm^R50X/R50X^* mouse model. Even though it was a promising therapy, no read-through induction was observed in any of these cells cultures with any of the RTAs tested, but further studies are needed to provide a better understanding of these drugs [241].

#### 2.3.7. GSD Type VI (GSD-VI; Hers Disease)

##### Introduction

GSD-VI (OMIM #232700) is caused by pathogenic mutations in both alleles of the *PYGL* gene (located on chromosome 14q21-q22 [86]), which encodes the liver isoform of glycogen phosphorylase, resulting in the absence of glycogen degradation in liver cells [87]. It was first reported by Henry-Gery Hers in 1959 [88], and nowadays has an estimated prevalence of 1/65,000 to 80,000 live births [86,89]. GSD-VI has variable severity and can present in infancy/early childhood with hepatomegaly, distended abdomen, growth retardation, mild hypoglycemia, and ketosis [87]. Some cases have been described of associated severe hypoglycemia, marked hepatomegaly, muscular hypotonia, and postpandrial lactic acid elevation [90,91]. Although previously believed to be a benign condition, recent studies have reported liver fibrosis and HCA in patients with GSD-VI [89,92].

***Treatment:*** Treatment is based on frequent small meals with a normal composition, although uncooked cornstarch can be used between meals and at bedtime to prevent hypoglycemic episodes and ketosis [93]. The main aim of nutrition therapy is to prevent the primary disease manifestations (hypoglycemia, ketosis and hepatomegaly) as well as the secondary complications (short stature, delayed puberty and cirrhosis) by improving metabolic homeostasis [87].

##### Mouse Model

The ***Pygl^-/-^ mouse model*** was developed by inserting a cassette containing the flippase recognition target (FRT) and loxP sequences in the intron between exons 2 and 3 of *Pygl* gene in order to disrupt *Pygl* mRNA expression [216]. *Pygl^-/-^* mice exhibited hepatomegaly, excessive hepatic glycogen accumulation, mild fasting hypoglycemia and elevated blood ketone bodies during prolonged fasting [216]. Additionally, *Pygl^-/-^* mice showed progressive accumulation of hepatic glycogen with aging and increased risk of liver damage and inflammation, along with collagen deposition [216].

#### 2.3.8. GSD Type VII (GSD-VII; Tarui Disease)

##### Introduction

GSD-VII (OMIM #232800) was first described by Japanese physician Seiichiro Tarui in 1965 [94]. It is caused by pathogenic mutations in both alleles of the *PFKM* gene (located to chromosome 12q13.11), which encodes for the muscle isoform of the phosphofructo-1-kinase enzyme (PFK-M). This enzyme catalyzes the conversion of fructose-6-phosphate to fructose-1.6-diphosphate and its absence therefore causes a blockade in glycolytic flux. In this regard, the typical clinical presentation is virtually indistinguishable from GSD-V. Minor clinical differences include more common report of nausea and vomiting accompanying the exercise-induced crises, a minor frequency of myoglobinuria episodes and a less common description of the second wind phenomenon [56]. However, an important difference between the two conditions is that high-carbohydrate meals or pre-exercise carbohydrate ingestion exacerbate exercise intolerance in patients with GSD-VII, as glucose cannot be used by muscle cells because (as opposed to McArdle disease) the metabolic blockade occurs downstream glucose uptake [56]. Thus, the importance of free fatty acids for muscle oxidative metabolism has been revealed in GSD-VII patients [95]. Interestingly, *PFKM* gene is also expressed in erythrocytes along with the *PYGL* gene [96,97]. An interesting feature of this disease, first recognized in 1980 [98], is the presence in muscle of abnormal polysaccharide (with polyglucosan-like characteristics) in addition to ‘normal’ glycogen [56]. Patients with GSD-VII are classified into four different subclasses according to their clinical manifestations: classical form, late on-set form, infantile form and haemolytic form [99]. The classical form is characterized by exercise intolerance, muscle contractures, pain, and sometimes after intense physical efforts, nausea and vomiting. Elevated CK levels, hyperuricaemia, reticulocytosis, and increased serum bilirubin are also prevalent features [100]. The *late on-set form* presents with contractures and myalgias in later life, although exercise ability is already low in childhood [100]. Patients with the infantile form presents with arthrogryposis congenita, generalized weakness, diverse signs of multisystem involvement including seizures, cortical blindness, cardiomyopathy and die within the first year of life [56,100]. The haemolytic form presents with hereditary non-spherocytic haemolytic anemia but with no muscle symptoms [100].

***Treatment:*** Currently, no specific treatment options exist for GSD-VII. However, ketogenic diet has been reported to be beneficial in one GSD-VII patient with alleviation of muscle pain, increased exercise tolerance and an improvement of working capacity and mechanical efficiency [254].

##### Animal Models

(a) Canine models

***English Springer Spaniel, American Cocker Spaniels, Whippet and mixed breed dogs*** with PFK-M deficiency due to a nonsense mutation in the *Pfkm* gene (c.2228A>G in exon 21; p.Trp743*) have been identified [217,218,219,220,221,222]. These dogs had chronic hemolytic anemia associated with acute hemolytic crises and hemoglobinuria as well as mild metabolic myopathy associated with exertion and a variable degree of muscle wasting [221,223,224]. However, muscle cramping and severe progressive myopathy occurred rarely. In the case of the Whippet dogs, cardiac abnormalities were also present [222]. The absence of severe muscle disease in these dogs resulted from the compensatory *Pfkl* gene expression in the canine muscles as well as from their high oxidative capacity [217]. On the other hand, the severe hemolytic anemia observed in this model, which is not a common trait in human GSD-VII patients, is explained by the different composition of the PFK-M and PFK-L isoforms in human and dog erythrocytes (i.e., PFK-M accounts for 40–50% of total enzyme expression in humans, vs. 80–90% in dogs).

Additionally, a missense point mutation (c.550C>T; p.Arg184Trp) in the *Pfkm* gene was described in four ***Wachtelhunds dogs* [225,226]**. These dogs presented with exercise intolerance, hemolytic anemia as well as pigmenturia.

(b) Mouse model

The ***Pfkm^-/-^ mouse model*** was developed by deleting the 5′ promoter region and exon 3, which contains the translation start codon. These mice presented an elevated mortality around weaning (about 60%), and also during early adulthood (around 3 to 6 months of age), and very few animals survived more than 1 year [227]. They also showed exercise intolerance, high glucose-6-phosphate levels in the skeletal muscle, increased expression of genes involved in oxidative metabolism and mitochondrial biogenesis (peroxisome proliferator-activated receptor-gamma coactivator (*Ppargc1a*), peroxisome proliferator activated receptor delta (*Ppard*), carnitine palmitoyltransferase 1, muscle isoform (*Cpt1m*), cytrate synthase (Cs) and *Ucp2* (uncoupling protein 2), upregulation of MHC-I and IIa, as well as of genes involved in glucose uptake (solute carrier family 2, facilitated glucose transporter member 4, also known as ‘Glut4′ (*Slc2a4*) and Hexoquinase 2 (*Hk2*)). Skeletal muscle fibers revealed very high subsarcolemmal and intermyofibrillar glycogen accumulation, which altered fiber morphology [227]. High glycogen accumulation was also observed in the diaphragm and intercostal muscles. *Pfkm^-/-^ mice* also showed hemolysis and increased erythropoiesis evidenced by pronounced reticulocytosis and splenomegaly [227].

#### 2.3.9. GSD Type XV (GSD-XV)

##### Introduction

GSD-XV (OMIM #613507) is an autosomal recessive disorder caused by pathogenic mutations in the *GYG1* gene (located to chromosome 3q24), which codes for glycogenin-1, the protein that forms the core of glycogen in skeletal and cardiac muscle. Glycogenin is a glycosyltransferase enzyme that catalyzes an auto-glucosylation reaction that generates a glucose polymer primer (of 10 residues, approximately) for glycogen synthesis [101]. Further chain elongation is performed by GS, and branches are introduced by the GBE. In humans and most mammals, there are two glycogenin isoforms: *GYG1*, which is expressed in several different tissues, and *GYG2*, which is predominantly expressed in the liver and in lower levels in the heart and pancreas [102,103]. As opposed to humans, rodents carry a single *Gyg* gene, which is expressed in all tissues [103,104]. The onset of disease in patients generally occurs between the 2nd and 5th decade of life, and normally presents with progressive, widespread muscle weakness and wasting. Cardiac involvement is rare, although when it is observed in patients is not normally accompanied by skeletal muscle affection. Conversely, patients with skeletal muscle affection did not have cardiac disease [105]. CK levels are usually normal or mildly elevated, and histological analyses do not reveal major muscle fiber degeneration [255]. The presence of polyglucosan bodies is a histopathological hallmark of this disease [101]. Interestingly, it has been observed that glycogenin-1 deficiency in patients does not preclude the formation of glycogen in the skeletal muscle as affected patients expressed significant amounts of glycogenin-2 in this tissue [105].

##### Mouse Model

The ***Gyg^-/-^ mouse model*** was developed by inserting a cassette containing FRT and loxP sequences in the intron between exons 2 and 3, and a loxP site in the intron between exon 4 and 5 of the *Gyg* gene in order to disrupt *Gyg* gene expression [228]. The number of pups per litter was very low and most of the *Gyg^-/-^* pups died shortly after birth due to cardiorespiratory failure [228]. Interestingly, these mice presented normal glycogen levels in liver and brain, while skeletal and cardiac muscle contained four and seven times more glycogen, respectively, than their WT counterparts [228]. These glycogen granules were larger than those from WT mice but with a normal degree of branching. With regards to exercise capacity, these mice showed poor treadmill performance coupled with an increase in isometric force generation in the soleus (but not in the EDL), indicating that the absence of glycogenin specifically modifies the performance of this oxidative muscle toward a more glycolytic type [228]. These results were confirmed by a reduction in oxygen consumption in the *soleus* muscle to levels comparable to those of fast-twitch muscles.

## 3. Critical Discussion

In the present work we have reviewed a total of 42 different animal models of GSD, including 26 genetically modified mouse models (Table 3), 15 naturally occurring models (encompassing quails, cats, dogs, sheep, cattle and horses) (Table 4), and one genetically modified zebrafish model. To our knowledge, this is the most complete list of GSD animal models ever reviewed. Importantly, when all these animal models are analyzed together, we can observe some common traits, as well as model specific differences, that would be overlooked if each model was only studied in the context of a given GSD. For example, the amount of glycogen levels in the skeletal muscle of the *Pygm^R50X/R50X^* mouse model (20 to 40 times higher than WT mice [209]) in comparison to patients (2–3 higher than healthy individuals [256]) has always been considered an inherent problem of this specific model, and one of the main and most important differences between affected mice and patients. However, in the context of all GSD mouse models, this is a common characteristic among them, as most models present higher glycogen levels than those found in patients, e.g., 6^neo^/6^neo^, Δ6/Δ6, Δ14^neo^/Δ14^neo^ and *Gaa^c.1826dupA^* (GSD-II), *Agl_EX5_^-/-^ Agl_EX32_^-/-^* and *Agl_Ex6-10_^-/-^* (GSD-III), and *Gbe1^ys/ys^* (GSD-IV) mouse models, with similar levels to those found in the *Pygm^R50X/R50X^* mice (>20 times higher levels than WT mice) (Table 3). These significant differences in glycogen content between GSD patients and mice might be related to the faster metabolic rates occurring in the later, leading to an accelerated accumulation of glycogen in the affected tissues. On the other hand, large animal models present similar glycogen levels to GSD patients, e.g., the McArdle ovine model glycogen levels are 2–5 times higher than WT cows [257]. Overall, sheep, cattle or horses (5 out of 15 of naturally-occurring GSD models) constitute a better model to reproduce human disease phenotypes than small models such as quails, cats and dogs (the remaining 10 out of 15 naturally-occurring GSD models) or rodents (25 out of 26 genetically modified GSD models), as they present closer similarities in organs and body size, muscle bulk and overall physio-pathologic and metabolic characteristics [258]. However, there are intrinsic difficulties in working with large animal models compared with small models, such as manipulation, phenotype characterization (e.g., exercise testing), breeding, housing costs and space availability, and the capacity to be shared among different research groups.

Besides the previously discussed glycogen levels, another common characteristic observed in different GSD mouse models is the occurrence of perinatal death, which has not been observed in the corresponding GSD patients. In this regard, perinatal death has been reported in *Gys1^-/-^* (GSD-0b), *G6pc^-^/^-^* (GSD-Ia), *Slc37a4^-/-^* (GSD-Ib), *Gbe1^-/-^* (GSD-IV), *Pygm^R50X/R50X^* (GSD-V), *Pfkm^-/-^* (GSD-VII) and *Gyg^-/-^* (GSD XV) mouse models (Table 3). From these, *Gys1^-/-^*, *Gbe1^-/-^*, *Pygm^R50X/R50X^* and *Gyg^-/-^* mouse models have high proportion of early neonatal death in pups, while in *G6pc^-^/^-^, Slc37a4^-/-^* and *Pfkm^-/-^* models, the death has been observed around the weaning period (Table 3). A possible explanation about these early birth death episodes might be related to the deficit of glycogen-driven glucose availability in affected tissues to meet basic energy requirements, as glycogen might play a crucial role in energy supply during the neonatal period due the low content of glucose in milk [259]. In the case of *Gys1^-/-^*, *Gbe1^-/-^*, *Pygm^R50X/R50X^* and *Pfkm^-/-^* mouse models, the skeletal muscle cannot obtain glucose from glycogen either because there is an absence of glycogen (*Gys1^-/-^*), an altered synthesis (*Gbe1^-/-^*), absence of degradation (*Pygm^R50X/R50X^* mice) or the incapability to obtain energy from glycolysis (*Pfkm^-/-^*). Interestingly, both *Gys1^-/-^* and *Pygm^R50X/R50X^* mice present instantaneous *rigor mortis*. On the other hand, in the *G6pc^-^/^-^* and *Slc37a4^-/-^* mouse models, glucose deficiency originates by the incapacity of the liver to liberate glucose to the blood (either originated from glycogen degradation or gluconeogenesis), thus causing a deficit in glucose provision to high demanding metabolic tissues such as the brain or the skeletal muscle. Premature death in adult animals has also been observed in *Gaa KO^DBA^* (GSD-II), *Agl_EX32_^-/-^* (GSD-III), *Gbe1^neo/neo^* (GSD-IV) and *Pygm^R50X/R50X^* (GSD-V) mouse models, three of them directly involved in either cytoplasmic or lysosomal glycogen degradation (*Pygm^R50X/R50X^*, *Agl_EX32_^-/-^* and *Gaa KO^DBA^*) (Table 3), indicating that glycogen as a source of energy might by critical for these mice beyond the neonatal and weaning periods. Whether these fatal outcomes can be explained by common physio-pathologic mechanisms among these models, or respond to model-specific characteristics is not clear and needs further research.

Besides these common traits within GSD animal models, we can also find differences not only between the different GSD models (as expected), but also within different models for a given GSD, e.g., between the *6^neo^/6^neo^*, Δ6/Δ6 and *Gaa KO^DBA^* GSD-II mouse models, *Agl_EX5_^-/-^, Agl_EX32_^-/-^, Agl_Ex6-10_^-/-^ and Agl_Ex6-10_^-/-^2* GSD-III mouse models or *Gbe1^neo/neo^*, *Gbe1^-/-^* and *Gbe1^ys/ys^* GSD-IV mouse models (Table 3). These differences might be partially explained by the different genetic modifications or strategies used to generate each model, although we might also not forget that the differences in the expertise and focus of research between groups, the diversity of strategies and protocols used for their characterization and the variety of animal housing and handling conditions (e.g., diet composition, number of animals per cage, number of cages per room…) among facilities, surely play a role in the heterogeneity of the reported data.

All these GSD animal models have been a very useful in order to test and evaluate potential therapeutic treatments at the preclinical stage, and some of them have been later translated and used in clinical practice. The most paradigmatic example is the case of ERT in Pompe patients, which was initially evaluated in quails, followed by the 6^neo^/6^neo^ mouse model, and is currently being used in Pompe patients, significantly improving their survival rates and quality of life. However, this is not always the case; quite often therapeutic approaches that have succeeded in the correction of the disease phenotype in animal models or derived cell cultures, but have subsequently failed in clinical trials. In the case of McArdle disease, VPA clearly diminished the glycogen amounts in skeletal muscle cultures derived from the *Pygm^R50X/R50X^* mouse model [213], however, it failed to produce any significant benefit in patients [82]. In this regard, it should be noticed that the selection of animal models for studying certain diseases is not always evidence based. Several considerations must be taken, including cost, housing, feeding, veterinary support, and a particular’s group familiarity with a model. Because of this, the animal model chosen for a study may not be suited for the clinical question under investigation. Furthermore, limitations of the mouse models include a short lifespan that curtails the effects of long term treatments. As an example, more than 100 vaccines against HIV-like viruses have demonstrated efficacy in animal models, however, none to date, have worked in humans [260]. In cancer research, the average rate of successful translation of animal research to human clinical trials is about 8% [261]. Taking all of this in consideration, the selection of animal models for studying certain diseases is not always easy, as they do not reproduce all of the phenotypic characteristics of a concrete disease. The careful selection of the most informative species for an animal model is still very important, but it also presents a unique challenge for investigators. In this regard, several considerations should be taken into account, such as the financial feasibility, previous experiments performed with a specific model, the different biological characteristics among different species and the available palette of imaging and molecular techniques available for a given specie [4].

To summarize, animal models usually do not reproduce all the characteristics of the human diseases, therefore, it is important to have the characterization of these animals and to take into consideration the representative traits and the deficiencies of these animal models, in order to be able to study, understand and to find a therapy that allows to improve the life of patients or cure the human diseases.

## 4. Conclusions

To conclude, this report showed the characteristics and the importance of animal models, particularly in the case of GSDs, summarized in:

- Although animal models show some differences with respect to their counterpart human disease phenotype, such as higher tissular glycogen accumulation or premature death (the latter not reported in some human GSDs), they recapitulate most of the characteristics of human disease. Consequently, researchers should take into consideration the specific phenotypic particularities of these animals when working with them.
- Animal models allow a deeper study of the features of the disease, since they allow to measure more parameters, to take more biopsies or to perform behavioral studies, methods that are quite invasive and they are not possible to test in humans (e.g., to obtain and compare biopsies of different muscles and liver and therefore to have a more accurate characterization of the disease).
- Animal models are a necessary preclinical step to evaluate the efficacy and safety of possible treatments and therapies before they are testing in humans. Even though not all the promising treatments in animals could be of benefit for GSDs patients, these animal models may be the main (if not the only) approach to develop new therapies for improving the lives of patients or curing GSDs.

## Figures and Tables

**Table 1:** List of the different GSD. Four of the GSD (GSD-IX, X, XII and XIII) do not have any animal model [17,18,19]. The different GSD types are in bold.

GSD Type/Name	Affected Tissue/Cells	Enzyme Deficiency	Gene Defect	Clinical Features	Animal Model	References
**0a**	Liver	Liver Glycogen synthase	*GYS2*	*Postprandial hyperglycaemia, fasting hypoglycemia, hyperketonemia.*	Yes	[12,16,20]
**0b**	Muscle	Muscle glycogen synthase	*GYS1*	*Cardiomyopathy, exercise intolerance.*	Yes	[21,22]
**Ia/Von Gierke disease**	Liver, kidney	Glucose-6-phosphatase (G6Pase)	*G6PC*	*Fasting hypoglycemia, hepatomegaly, lactic acidemia, hypertriglyceridemia, hyperuricemia and growth retardation.*	Yes	[15,23,24,25,26,27,28]
**Ib**	Liver, kidney, leukocytes	Glucose-6-phosphate transporter T1 (G6PT)	*SLC37A4*	*In addition of Ia: neutropenia and neutrophil dysfunction that predispose to recurrent infections.* *Kidney and renal disease, splenomegaly and hepatocellular adenoma.*	Yes	[12,16,29,30]
**II/Pompe disease**	All (Liver, skeletal muscle, leukocytes, fibroblast, amniocytes)	α-1-4-glucosidase (Acid maltase/GAA)	*GAA*	*Lysosomal storage disease. Cardiomyopathy, respiratory failure, muscular weakness, hypotonia.*	Yes	[12,13,16,31,32,33,34,35,36,37,38,39,40,41,42,43,44,45,46,47,48]
**IIIa/Cori disease**	Liver, skeletal and cardiac muscle	Glycogen debranching enzyme (GDE)	*AGL*	*Hepatomegaly, fasting hypoglycemia, hyperlipidemia, muscle weakness, growth retardation, myopathy and cardiomyopathy. Osteoporosis and polycystic ovaries.*	Yes	[49,50,51,52,53,54,55,56,57,58,59,60,61,62,63,64,65]
**IIIb**	Liver	Glycogen debranching enzyme (GDE)	*AGL*	*As type IIIa but no muscle weakness.*	Yes	
**IIIc**	Liver, skeletal and cardiac muscle	Glycogen debranching enzyme (GDE)	*AGL*	*Selective loss of glucosidase.*	Yes	
**IIId**	Liver, skeletal and cardiac muscle	Glycogen debranching enzyme (GDE)	*AGL*	*Selective loss of transferase.*	Yes	
**IV/Andersen disease**	Liver, skeletal muscle	Glycogen branching enzyme (GBE)	*Gbe1*	*Altered growth, cognitive impairment, portal hypertension, hepatosplenomegaly and progressive liver cirrhosis. Myopathy resembling muscular dystrophy with difficulty walking and proximal limb weakness.*	Yes	[16,23,66]
**V/McArdle disease**	Skeletal muscle	Muscle glycogen phosphorylase enzyme	*PYGM*	*Exercise intolerance, muscle cramps, pain, crisis of early fatigue and contracures. Rhabdomyolysis (50% of cases), hyperckemia, myoglobinuria may lead to acute renal failure. Second-wind phenomenon.*	Yes	[67,68,69,70,71,72,73,74,75,76,77,78,79,80,81,82,83,84,85]
**VI/Hers disease**	Liver	Liver Glycogen phosphorylase	*PYGL*	*Hepatomegaly, growth retardation, hypoglycemia, ketosis as well as liver fibrosis.*	Yes	[86,87,88,89,90,91,92,93]
**VII/Tarui disease**	Skeletal muscle, leukocytes	Muscle Phosphofructo-1-kinase enzyme	*PFKM*	*Exercise intolerance, muscle cramps, pain, nausea and vomiting. Hyperckemia, hyperuricaemia, reticulocytosis, and increased serum bilirubin.* *Haemolytic anemia.*	Yes	[56,94,95,96,97,98,99,100]
**IX**	Liver, Muscle	Phosphorylase-b-kinase	*PHKA, PHKA2, PHKAB, PHKAG1, PHKG2, PHKD*	*Growth retardation, hepatomegaly. Mild hypertriglyceridemia, hypercholesterolemia, and elevated serum transaminase levels may be present.*	No	[12,14]
**X**	Skeletal muscle	Muscle phosphoglycerate mutase	*PGAM2*	*Muscle cramping, rhabdomyolysis, and myoglobinuria*	No	[17]
**XII**	Skeletal muscle and leukocytes	Aldolase A	*ALDOA*	*Hemolytic anemia, rhabdomyolysis and myoglobinuria.*	No	[18]
**XIII**	Skeletal muscle	β-enolase	*ENO3*	*Exercise intolerance, myalgias and mildly hyperckemia.*	No	[19]
**XV**	Skeletal and cardiac muscle	glycogenin-1	*GYG1*	*Muscle weakness and wasting and cardiac arryhthmias associated with PG accumulation.*	Yes	[101,102,103,104,105]

**Table 2:** Complete list of GSD animal models. * Treatment tested in cell cultures derived from the mouse model.

GSD Type	Animal Model	Naturally Occurring	Specific Model	Therapies Evaluated	References
**GSD-0b**	Murine model	No	*Gys1^-/-^*	Not reported	[106,107,108]
**GSD-Ia/Von Gierke**	Canine model	Yes	*Maltase dog*	Gene therapy	[109,110,111,112,113,114,115]
		Yes	*Maltase-Beagle dog*	Gene therapy	[111,112,113,114,115,116]
	Murine model	No	*G6pc^-/-^*	Glucose therapy Gene therapy	[112,117,118,119,120,121,122,123,124,125,126,127]
		No	*L-G6pc^-/-^*	Gene therapy RNAi approach	[128]
		No	*K-G6pc^-/-^*	Not reported	[129]
		No	*I-G6pc^-/-^*	mRNA therapy Gene therapy	[130]
**GSD-Ib**	Murine model	No	*Slc37a4^-/-^*	Gene therapy	[131,132,133,134]
		No	*TM-Slc37a4^-/-^*	Not reported	[135]
**GSD-II Pompe**	Cattle models	Yes	*Shorthorn cattle*	Not reported	[136,137]
		Yes	*Brahman cattle*	Not reported	[138,139,140,141]
	Canine model	Yes	*Lapphund dog*	Gene therapy	[142,143,144,145]
	Murine model	No	*6^neo^/6^neo^*	ERT Gene therapy	[146,147,148,149,150,151,152,153,154,155,156,157,158,159,160,161,162,163,164,165,166,167,168,169,170,171,172,173,174,175,176,177,178,179,180,181,182]
		No	*AD-6^neo^/^6neo^*	Not reported	[183,184]
		No	*AD2-6^neo^/6^neo^*	Not reported	[185]
		No	Δ*6/*Δ*6*	Not reported	[146,150]
		No	*13^neo^/13^neo^*	Not reported	[186]
		No	Δ*14^neo^/*Δ*14^neo^*	Not reported	[150]
		No	*Gaa^KODBA^*	Not reported	[151]
		No	*Gaa^c1826dupA^*	Not reported	[183]
	Quail models	Yes	*Japanese quails*	ERT	[187,188,189,190]
**GSD-III/Cori**	Canine model	Yes	*Curly-coated retrievers (CCR)*	Not reported	[191,192,193]
	Murine model	No	*Agl_EX5_^-/-^*	RNAi approach	[49]
		No	*Agl_EX32_^-/-^*		[194]
		No	*Agl_EX6-10_^-/-^*	Gene therapy	[195]
		No	*Agl_EX6-10_^-/-^2*	Gene therapy	[196]
**GSD-IV/Andersen**	Horse model	Yes	*American Quarter Horse foals (AQHs)*	Not reported	[197]
	Cat Model	Yes	*Norwegian forest cats*	Not reported	[198]
	Murine Model	No	*Gbe1^neo^/^neo^*	Not reported	[199]
		No	*Gbe1^-/-^*	Not reported	[199]
		No	*Gbe1^ys/ys^*	Gene therapy	[200,201]
**GSD-V/McArdle**	Bovine model	Yes	*Charolais cattle*	Not reported	[202,203]
	Ovine model	Yes	*Merino sheep*	VPA treatment Gene therapy	[204,205,206,207]
	Murine model	No	*Pygm^p.R50X/p.R50X^*	VPA treatment Read-through * Gene therapy	[208,209,210,211,212,213,214]
	Zebrafish model	No	*N.A.*	Not reported	[215]
**GSD-VI/Hers**	Murine model	No	*Pygl^-/-^*	Not reported	[216]
**GSD-VII/Tauri**	Canine models	Yes	*English Springer Spaniel*	Not reported	[217,218,219,220,221,222,223,224]
		Yes	*American Cocker Spaniels*	Not reported	[217,218,219,220,221,222,223,224]
		Yes	*Whippet*	Not reported	[217,218,219,220,221,222,223,224]
		Yes	*Wachtelhunds*	Not reported	[225,226]
	Murine model	No	*Pfkm^-/-^*	Not reported	[227]
**GSD-XV**	Murine model	No	*Gyg^-/-^*	Not reported	[228]

**Table 3:** Main characteristics of the different GSD mouse models. Abbreviations. ALP: alkaline phosphatase, ALT: alanine transaminase, AST: aspartate transaminase, CPK: creatine phosphokinase, diaph: diaphragm, f.i: fold increase, HCA: hepatocellular adenoma, N.A: not applicable, Skm: skeletal muscle.

Glycogenosis	Model	Major Organs Affected	Growth Impairment	Blood Analyses	Glycogen Content	Impaired Exercise Capacity	Premature Death	% of Premature Death/Cause	References
0b	*Gys1^-/-^*	Heart	Yes	Hypolactatemia	Absence (skeletal muscle, heart)	Endurance (fasting)	Yes/perinatal	~90%/abnormal cardiac development	[106,107,108]
Ia	*G6pc^-/-^*	Liver, kidney	Yes	Hypoglycemia, hyperlipidemia, hyperuricemia	Increased (~20 and ~30 f.i in liver and kidney, respectively)	Not reported	Yes/~weaning	~90%/hypoglycemia	[112,117,118,119,120,121,122,123,124,125,126,127]
	*L-G6pc^-/-^*	Liver	No	Hypertriglyceridemia, hyperuricemia, hypercholesterolemia, hyperlactacidemia	Increased (~2–2.5 f.i in liver)	Not reported	No	N.A.	[128]
	*K-G6pc^-/-^*	Kidney	No	Hyperuricemia	Increased (~22 f.i in kidney)	Not reported	No	N.A.	[129]
	*I-G6pc^-/-^*	Intestine	No	None reported	None reported	Not reported	No	N.A.	[130]
Ib	*Slc37a4^-/-^*	Liver, kidney, bone marrow, spleen	Yes	Hypoglicemia, hyperlipidemia, hyperuricemia, hyperlactacidemia, neutropenia, leucopenia	Increased (liver, kidney)	Not reported	Yes/~weaning	~90%/hypoglycemia	[131,132,133,134]
	*TM-Slc37a4^-/-^*	Liver, kidney, bone marrow, spleen	No	Hypertriglyceridemia, hypercholesterolemia, hyperalbuminemia, neutropenia, Increased AST	Increased (~6.5 and ~2.5–5 f.i in kidney and liver, respectively)	Not reported	No	N.A.	[135]
II	*6^neo^/6^neo^*	Skm, heart, diaph	No	Not reported	Increased (~45, 17, 16 f.i in heart, quad and gast, respectively). Also in diaph	Yes	No	N.A.	[146,147,148,149,150,151,152,153,154,155,156,157,158,159,160,161,162,163,164,165,166,167,168,169,170,171,172,173,174,175,176,177,178,179,180,181,182]
	*AD-6^neo^/6^neo^*	Skm, heart, diaph	Yes	Not reported	Increased (~45, 11, 10 f.i in heart, quad and gast, respectively). Also in diaph	Yes	Yes	Not reported/respiratory failure	[183,184]
	*AD2-6^neo^/6^neo^*	Skm, heart, diaph	Not reported	Not reported	Increased (~45, 8, 5.5 f.i in heart, quad and gast, respectively). Also in diaph	Not reported	Not reported	Not reported	[185]
	Δ*6/*Δ*6*	Skm, heart, diaph	No	Not reported	Increased (~360 and ~42 f.i in diaph and skm, respectively). Also in heart and brain	No	No	N.A.	[146,150]
	*13^neo^/13^neo^*	Skm, heart	No	Not reported	Increased (skeletal muscle, heart)	Not reported	No	N.A.	[186]
	Δ*14^neo^/*Δ*14^neo^*	Skm, heart, brain	No	Not reported	Increased (~320 and ~41 f.i in diaph and skm, respectively). Also in heart and brain	Yes	No	N.A.	[150]
	*Gaa KO^DBA^*	Skm, heart, diaph	No	Not reported	Increased (skm, heart diaph)	Yes	Yes/within first 6 months	~50%/respiratory failure	[151]
	*Gaa^c.1826dupA^*	Skm, heart, diaph	Yes	Not reported	Increased (~185 and ~28 f.i. in heart and gast, respectively)	Yes	No	N.A.	[183]
III	*Agl_EX5_^-/-^*	Liver, Skm, heart	No	Hypoglycemia, Increased AST, ALT, ALP and CPK	Increased (~200, ~50, ~25, ~22 f.i in liver, diaph, heart and gast, respectively)	Yes	No	N.A.	[49]
	*Agl_EX32_^-/-^*	Liver, Skm, heart	No	Hypoglycemia, Increased ALT, AST, ALP	Increased (~26, ~22, ~2.5 f.i. in heart, skm, liver respectively)	Yes	Yes/within first year	Unknown	[194]
	*Agl_EX6-10_^-/-^*	Liver, Skm, heart, brain	No	Hypoglycemia	Increased (~8, ~3, ~3 f.i. in skm, liver, heart, respectively)	Yes	No	N.A.	[195]
	*Agl_EX6-10_^-/-^ 2*	Liver, Skm, heart, brain	No	Increased ALT	Increased (~150, ~50, ~30 f.i. in liver, skm, heart respectively)	Yes	No	N.A.	[196]
IV	*Gbe1^neo^/^neo^*	Liver, Skm, heart, brain	No	Hypoglycemia (slight)	Increased (liver, skm)	Not reported	Yes/within first 11 months	~100%/unknown	[199]
	*Gbe1^-/-^*	Liver, Skm, heart	N.A	None reported	Reduced (skm)	Not reported	Yes/soon after birth	~100%/unknown	[199]
	*Gbe1^ys^/^ys^*	Liver, Skm, heart, brain	No	Hypoglycemia (slight), hyperCKemia, increased ALT and AST	Increased (~90, ~32, ~17, ~1.5, heart, skm, brain and liver, respectively	Yes	Yes/before 20 months	~100%/unknown	[200,201]
V	*Pygm^R50X/R50X^*	Skm	No	HyperCKemia, hyperammonemia, Hypolactatemia, hypoglycemia	Increased (~40, ~20 and ~15 f.i in TA, quad and gast, respectively)	Yes	Yes/perinatal and post-weaning	~85%/unknown	[208,209,210,211,212,213,214]
VI	*Pygl^-/-^*	Liver	No	Hypoglycemia, hyperketonemia, increased ALT and AST	Increased (~60 f.i in liver)	Not reported	No	N.A.	[216]
VII	*Pfkm^-/-^*	Skm, heart, diaph, spleen	No	Hypolactatemia, hyperbilirubinemia	Increased (~8 f.i in diaph). Also increased in skm and heart	Yes	Yes/around weaning	60%/unknown	[227]
XV	*Gyg^-/-^*	Skm	No	Not reported	Increased (7 and 4 f.i. in heart and skm)	Decreased aerobic, increased anaerobic	Yes/perinatal	~85%/cardio-respiratory failure	[228]

**Table 4:** Main characteristics of the different naturally-occurring GSD models.

GSD Type	Natural Occurring	Mutation	Enzyme Activity	Biochemical and Clinical Features	Glycogen Accumulation	Life Expectancy	References
GSD-I	Maltese canine model	*G6pc* p.121 M>I	G6P Levels reduced	Hypoglycemia, hepatomegaly and necropsy. Failure to thrive, liver and Kidney pale.	Hepatocytes and kidneys	5–8 weeks	[109,110,111,112,113,114,115,116]
GSD-II Pompe	Shorthorn cattle	*Gaa* c.2454_2455delCA	Loss GAA ativity	Muscle weakness, respiratory distress, congestive heart failure.	Skeletal muscle, heart, and central nervous tissue	12 months	[136,137]
	Brahman cattle	c.1057_1058delTA	Loss GAA ativity	Progressive muscular weakness	Cytoplasmic vacuolation in brain, spinal cord, skeletal muscle, myocardium and Purkinje fibers	12 months	[138,139,140,141]
	Lapphund dogs	*Gaa* c.2237G>A p.W746X	Absence GAA activity	Megaesophagus, exercise intolerance, and recurrent emesis.	Vacuoles in heart, skeletal muscle, and smooth muscle	10–18 months	[141,142,143,144,145]
	Japanese quails	Defect in GAA protein maturation and processing	Absence GAA activity	Progressive muscle weakness and myopathy	Vacuoles and glycogen depots in muscle and cardiac tissue. Pectoralis most affected tissue.	18 months	[187,188,189,190]
GSD-III Cori	Curly-coated retrievers (CCR)	*Gaa* c.4223delA	GDE Levels reduced	Progressive muscle damage and hepatomegaly, precense of nudles, cirrhosis. High ALT, AST and ALP levels, slightly high CPK levels.	Cardiomyocytes, gradual increase in Liver and skeletal muscle	11–12 years	[191,192,193]
GSD-IV Andersen	American Quarter Horse foals (AQHs)	*Gbe1* p.Y34X	GBE amount and activity reduced	Stillbirth, transient flexural limb deformities, respiratory or cardiac failure.	Skeletal muscle, liver and central nervous system	1–18 weeks	[197]
	Norwegian forest cats	*Gbe1* g. IVS11 + 1552_IVS12-1339 del6.2 kb ins334 bp	Absence GBE activity	Skeletal muscle atrophy, progressive neurological decline, generalized muscle tremors.	Skeletal muscle, Liver, cardiatic muscle, neurvous system	1–13 months	[198]
GSD-V McArdle	Charolais cattle	*Pygm* p.W489R	GP-MM amount and activity reduced	Exercise intolerance, rhabdomyolysis, high CK levels and myoglobinuria.	Skeletal Muscle	Not reported	[202,203]
	Merino sheep	missense mutation in the 3′ splice acceptor site of intron 19 of *Pygm*	Absence GP-MM activity	Exercise intolerance, rhabdomyolysis	Skeletal Muscle	Not reported	[204,205,206,207]
GSD-VII Tarui	English Springer Spaniel	*Pfkm* c.2228 G>A	Absence of PFKM activity	Chronic hemolytic anemia, hemolytic crises, hemoglobinuria, hyperbilirubinuria, moderately increased serum creatine kinase activity, muscle wasting.	Skeletal muscle	11 years	[217,218,219,220,221,222,223,224]
	American Cocker Spaniels	*Pfkm* c.2228 G>A	Absence of PFKM activity		Skeletal muscle	11 years	[217,218,219,220,221,222,223,224]
	Whippet	*Pfkm* c.2228 G>A	Absence of PFKM activity	Mimics English Springer Spaniel symptoms but present cardicac and muscular abnormalities	Skeletal muscle	4 years	[217,218,219,220,221,222,223,224]
	Wachtelhunds	*Pfkm* c.550C>T	PFKM activity reduced	Exercise intolerance, hemolytic anemia, pigmenturia	Skeletal muscle	4 years	[225,226]

**Table 5:** Description of the different therapeutic approaches evaluated in GSD models. Abbreviations. ERT: Enzyme replacement therapy, VPA: Sodium valproate, RTAs: read-through agents, RSV: Rous sarcoma virus, CMV/CB: Citomegalovirus/chicken β-actin, MCK: muscle creatine kinase, SFFV: spleen focus-forming virus, pfu: plaque forming units, IU: infectius units, vg: viral particles. * Treatment tested in cell cultures derived from the mouse model.

Type	Specific Model	Therapies Evaluated	Vector/Compound	Promoter	Main Effect	References
**GSDIa Von Gierke disease**	Canine: Maltase-Beagle dog	Gene therapy	AAV2-G6pc	Mouse albumin promoter	G6Pase activity sgnificantly increased and liver glycogen content was significantly reduced.	[111]
			AAV2/8 vector	Human G6Pc promoter (−298 to +128)	G6Pase activity was increased in liver and glycogen content reduced. Effects decrease with time.	[112,113]
			HDAd-G6Pc	Human apoAI promoter	Increase in liver G6Pase activity and a decrease in liver glycogen content, long-term complications such as HCA.	[114]
	Murine: G6pc^-/-^	Glucose therapy	Glucose 10%	N.A.	Failed to sustain survivla beyond weaning (21 days).	[119]
		Gene therapy	Ad-G6pc	RSV promoter	19% restoration of hepatic G6Pase activity and improved survival and growth. G6Pase did not transduce to kidney.	[119,120,121]
					~16% restoration of hepatic G6Pase activity. G6Pase did not transduce to kidney.	[119,120,121]
			AAV2-G6pc	CMV/CB promoter	Tretament failed to improve the survival of treated mice after weaning due to the delayed kinetics of rAAV-mediated transgene expression	[120]
			Ad-G6pc	RSV promoter	~33% restoration of G6Pase activity at 8.5 months. No hepathomegaly or nephromegaly was seen.	[120]
			+	+		
			AAV2-G6pc	CMV/CB promoter		
			AAV8-G6pc	CMV/CB promoter	~20% restoration of G6Pase activity in the liver but almost null activity in the kidneys.	[122]
			AAV1-G6pc	CMV/CB promoter	~10% restoration of G6Pase mantained for 57 weeks in the liver and 7% in kidneys. No hepatomegaly or nephromegaly was observed.	[122]
			AAV8-G6pc	Canine G6Pc promoter	25% G6Pase activity up to 7 months, correction of growth retardation and fasting hypoglycemia, reduction of liver glycogen content.	[123]
			AAV8-G6pc	Human G6Pc promoter (−298 to +128)	Hepatic G6Pase activity was recovered to almost normal levels. Lost of effects at 26w.	[112]
			AAV8-G6pc	Human G6Pc promoter (−2864 to −1)	Complete normalization of G6Pase activity, glycemia and glycogen and lipid storage in liver.	[124,125]
	Murine:L-G6pc^-/-^	RNAi approach	Gys2 siRNA	N.A.	Hepatic glycogen accumulation reduced due to the reduction in Gys2 mRNA levels.	[240]
**GSDIb**	Murine: Slc37a4^-/-^	Gene therapy	Ad-SLC37A4	Not reported	G6PT levels restored in liver, bone marrow and spleen. Hypoglycemia was restored and glycogen accumulation was reduced but some premature deaths were observed.	[132]
			AAV8-SLC37A4	CMV/CB promoter	Transgene was primarily delivered to liver but there was long-term complications as HCA.	[133]
			AAV8-SLC37A4	Human G6Pc promoter	Correction of the metabolic abnormalities with a great efficacy.	[134]
			AAV8-SLC37A4	Human SLC37A4 promoter	Correction of the metabolic abnormalities with less efficacy than the human G6Pc promoter.	[134]
**GSDII Pompe Disease**	Japanese quail	ERT	rhGAA	N.A.	Reduced glycogen content and increase of GAA activity in all tissues.	[189]
	Murine: 6^neo^/6^neo^	ERT	rhGAA + mannose-6-P	N.A.	Improvement of muscular affection with mannose-6-phosphate helping the uptake by skeletal muscle cells.	[152]
			rhGAA + glycosylation independent lysosomal targeting tag	N.A.	Improvement of muscular affection with the glycosilation independent lysosomal targeting target leading to an improvement of its lysosomal delivery.	[153]
			rhGAA + anti-B cell activating factors drugs	N.A.	Improvement of muscular affection and increase of GAA activity.	[154]
		Gene therapy	AAV2-GAA	CMV/CB promoter	Activity of GAA almost normal for at least 6 weeks, reduced glycogen accumulation and preserved skeletal muscle force, but the effect is confined to injected muscle.	[155]
			AAV2/6-GAA	MCK promoter/enhancer	Expression of GAA at approximately 100-fold increase significantly reducing the glycogen content in the injected muscle.	[156]
			AAV2/1-GAA	CMV promoter	Almost normal GAA activity levels, reduction in glycogen accumulation and a significant improvement in the contractility of the injected diaphragm.	[157]
			AAV1-GAA	CMV promoter	Persistent transgene expression in the injected muscles with muscle improvement and glycogen clearance by crosscorrection.	[158]
			AAV9-GAA	CMV promoter		
			Different AAV-GAA	Muscle promoters	Different studies showed an improvement of injected muscle with some glycogen correction.	[156,159,160,161,162,163,164,165]
			Different AAV-GAA	Liver promoters	Efficient liver transduction of the gene with glycogen clearance in different muscles by crosscorrection.	[147,148,152,166,167,168,169,170,171,172]
			AAV9-GAA	Tandem liver/muscle promoter	Persistent therapeutic efficacy allowing a crosscorrection in different target tissues.	[173]
			AAV8-GAA			
			AAV9/3-GAA	Neuron-specific promoter	Glycogen reduction in CNS but not in muscle.	[174]
			AAV9-GAA	CMV/CB promoter	Glycogen reduction in CNS and in heart.	[175]
			AAVrh10-GAA	CMV/CB promoter		
		Pharmacological chaperon AT220	rhGAA + AT2220	N.A.	Significant increase of rhGAA levels in plasma and a glycogen reduction in heart and skeletal muscles.	[176,177]
		Transplantation of lentiviral vector	Codon-optimized GAA lentiviral vector	SFFV promoter	Sustained GAA activity in heart and skeletal muscle and glycogen accumulation near normal levels.	[178,179,180]
		Leucine supplementation	Leucine	N.A.	Chronic leucine feeding increased activity and running capacity with reduced glycogen accumulation.	[181]
		Satellite cell activation	BaCl_2_ or cardiotoxin	N.A.	Muscular regeneration but unable to repair the damage.	[182]
**GSDIII Cori Disease**	Murine: Agl-/-	RNAi approach	Gys2 siRNA	N.A.	Silenced hepatic Gys2 expression prevented glycogen synthesis and accumulation and nodule development.	[240]
	Murine: Agl_EX6–10_^-/-^	Gene therapy	AAV-GAA vector	CMV/CB promoter	Dicrease in glycogen levels in liver but not in skeletal muscle. GDE protein levels dectected in heart and skeletal muscle, but not in the liver. Not correction of Hepatomegaly.	[195]
			+			
			AAV9-Agl			
	Murine: Agl_EX6-10_^-/-^ 2	Gene therapy	AAV-Pullulanase	N.A.	Reduced glycogen content and improved liver and muscle functions.	[196]
**GSDIV Andersen Disease**	Murine: Gbe1^ys^/^ys^	Gene therapy	AAV9-GBE	CMV/CB promoter	GBE activity increased and glycogen content was reduced in heart, skeletal muscle, brain and liver.	[201]
**GSDV McArdle Disease**	Ovine: Merino sheep	Compounds inducing re-expression of the fetal Pygb/Pygl isoforms	Notexin (5 µg/mL)	N.A.	Re-expression of non-muscular fetal isoforms and reduction of glycogen content.	[205,206]
			VPA (0.5 g/30 mL)	N.A.	Insufficient increment of non-muscular fetal isoforms.	[206]
		Gene therapy	AdV5-PYGM	RSV or CMV/CB promoter	Increase of glycogen phosphorilase and reduction of glycogen accumulation in the site of injection, decreasing with time.	[207]
			+			
			AAV2-PYGM			
	Murine:PYGM ^p.R50X/p.R50X^	Compounds inducing re-expression of the fetal *Pygb*/*Pygl* isoforms	VPA *	N.A.	Increased expression of *Pygb* mRNA levels in myotubes.	[213]
			VPA	N.A.	No significant changes in skeletal muscle glycogen content or *Pygb* expression were observed.	Unpublished data
		Gene therapy	AAV8-PYGM	CMV promoter	Pygm expression with reduced muscle glycogen content and voluntary activity restored to normal levels.	[214]
		Read-through treatment	RTAs *	N.A.	No read-through induction was observed with any of the agents used.	[241]
